# Cassette recruitment in the chromosomal Integron of *Vibrio cholerae*

**DOI:** 10.1093/nar/gkab412

**Published:** 2021-05-28

**Authors:** Claire Vit, Egill Richard, Florian Fournes, Clémence Whiteway, Xavier Eyer, Delphine Lapaillerie, Vincent Parissi, Didier Mazel, Céline Loot

**Affiliations:** Institut Pasteur, Unité Plasticité du Génome Bactérien, CNRS UMR3525, Paris, France; Sorbonne Université, Collège doctoral, F-75005 Paris, France; Institut Pasteur, Unité Plasticité du Génome Bactérien, CNRS UMR3525, Paris, France; Sorbonne Université, Collège doctoral, F-75005 Paris, France; Institut Pasteur, Unité Plasticité du Génome Bactérien, CNRS UMR3525, Paris, France; Institut Pasteur, Unité Plasticité du Génome Bactérien, CNRS UMR3525, Paris, France; Institut Pasteur, Unité Plasticité du Génome Bactérien, CNRS UMR3525, Paris, France; CNRS, UMR5234, Fundamental Microbiology and Pathogenicity laboratory, University of Bordeaux. Département de Sciences Biologiques et Médicales, Bordeaux, France; Viral DNA Integration and Chromatin Dynamics Network (DyNAVir), France; CNRS, UMR5234, Fundamental Microbiology and Pathogenicity laboratory, University of Bordeaux. Département de Sciences Biologiques et Médicales, Bordeaux, France; Viral DNA Integration and Chromatin Dynamics Network (DyNAVir), France; Institut Pasteur, Unité Plasticité du Génome Bactérien, CNRS UMR3525, Paris, France; Institut Pasteur, Unité Plasticité du Génome Bactérien, CNRS UMR3525, Paris, France

## Abstract

Integrons confer a rapid adaptation capability to bacteria. Integron integrases are able to capture and shuffle novel functions embedded in cassettes. Here, we investigated cassette recruitment in the *Vibrio cholerae* chromosomal integron during horizontal transfer. We demonstrated that the endogenous integrase expression is sufficiently triggered, after SOS response induction mediated by the entry of cassettes during conjugation and natural transformation, to mediate significant cassette insertions. These insertions preferentially occur at the *attIA* site, despite the presence of about 180 *attC* sites in the integron array. Thanks to the presence of a promoter in the *attIA* site vicinity, all these newly inserted cassettes are expressed and prone to selection. We also showed that the RecA protein is critical for cassette recruitment in the *V. cholerae* chromosomal integron but not in mobile integrons. Moreover, unlike the mobile integron integrases, that of *V. cholerae* is not active in other bacteria. Mobile integrons might have evolved from the chromosomal ones by overcoming host factors, explaining their large dissemination in bacteria and their role in antibioresistance expansion.

## INTRODUCTION

Mobile Genetic Elements (MGE) widely contribute to the evolution of bacterial genomes, notably by conveying adaptive traits such as the ability to resist to antibiotic treatments (1). This can have dramatic consequences, especially when occurring in pathogenic bacteria. Integrons are genetic structures that are considered as major contributors in the rise of multiple antibiotic resistance in Gram-negative bacteria. These genetic systems were discovered in the late 80s and described as platforms involved in the capture, stockpiling and expression of antibiotic resistance genes, embedded in structures termed ‘cassettes’ (2). These were later referred to as Mobile Integrons (MIs) because of their associations with transposable elements and conjugative plasmids. Larger integrons located on bacterial chromosomes were discovered later, the superintegron of *Vibrio cholerae* being the first identified (3). This superintegron is located on chromosome 2 of *V. cholerae* and contains about 180 gene cassettes coding mainly for proteins with no homologs in the databases or for proteins of unknown functions. In contrast to their mobile counterparts and to refer to their location, such structures are termed Sedentary Chromosomal Integrons (SCIs). They are common features of bacterial genomes from the *Vibrio* genus and are generally distributed in several genomes of β- and γ-proteobacteria (4,5). These large SCIs were proposed to be the ancestors of MIs (6) and the stockpiling capacity from both types of integrons suggests that they may have distinct but complementary roles (7). Indeed, large SCIs such as those found in genomes of *Vibrio* species, could constitute a reservoir of gene cassettes that can be captured and spread by MIs (7,8).

**Figure 1.**
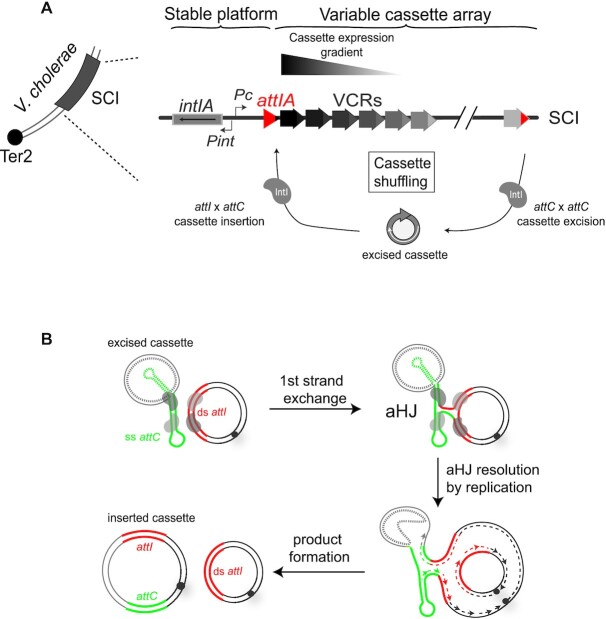
The integron. (**A**) The integron system in *Vibrio cholerae*. *V. cholerae* sedentary chromosomal integron is located on the second chromosome close to the termination site, Ter2. The four components of the integron stable platform are shown: the integrase expressing gene, *intIA*, the two promoters, P_C_ and P_int_ and the *attIA* recombination site (red triangle). The variable cassette array contains a large number of cassettes, which are represented by small arrows. Their expression level is reflected by the colour intensity of each arrow. Only the first cassettes of the array are expressed, and the subsequent ones can be seen as a low-cost cassette reservoir. Upon expression of the integrase (grey forms) cassette shuffling can occur through cassette excision (*attC* × *attC*) and integration in the first position in the array (*attIA* × *attC*). (**B**) Integron cassette insertion in an *attI* site. Recombination between the double-stranded *attI* site (bold red lines) and a single-stranded bottom *attC* site (green lines) ending a cassette is shown. Since we do not exactly know the nature of the cassettes (ss or ds), the top strand of the *attC* site is represented as a dotted line. The synaptic complex comprises both *att* sites bound by four integrase monomers (grey ovals). One strand from each *att* site is cleaved and transferred to form an atypical Holliday junction (aHJ). aHJ resolution implies a replication step. The origin of replication is represented by a grey circle and the newly synthesized leading and lagging strands by dashed lines. Both products are represented: the initial substrate resulting from the top strand replication, and the reactional product containing the inserted cassette and resulting from the bottom strand replication.

Both types of integrons are composed of a stable platform and a variable cassette array. The platform contains a gene coding for a tyrosine-recombinase, IntI, the *attI* recombination site and the resident promoter P_C_ oriented towards the variable cassette array (Figure 1A). The cassette array contains a pool of gene cassettes generally composed of single promoter-less genes (coding sequence, CDS), so that their expression relies on the P_C_ promoter. Starting from P_C_, their expression gradually decreases in the array (9) (Figure 1A). Then, the gene cassettes that are located close enough to the P_C_ promoter are the only ones to be expressed, except for those that contain their own promoter (10–13). The integron system represents a low-cost and modular reservoir of adaptive functions for their host bacteria. Each gene cassette is flanked by an *attC* recombination site that is recognized and recombined by the integrase. The latter catalyzes the different reactions that lead to cassette mobilization. By recombining *attI* and *attC* sites, the integrase allows the recruitment of cassettes downstream of the P_C_ promoter and express them (Figure 1A). By recombining two consecutive *attC* sites, the integrase ensures the excision of one cassette. Subsequent excision and integration of a circular cassette in the first position of the array, constitute a way for a previously silent gene to be expressed (Figure 1A).

A key feature of the integron is the ability of the integrase to recombine both single-stranded DNA (*attC* site) and double-stranded DNA (*attI* site) depending on their structure and sequence respectively (Figure 1B) (14,15). Indeed, each integrase recognizes the sequence of its cognate *attI* site (16,17). In contrast, the integrase does not recognize the sequence of *attC* sites, but rather the structure of the ss folded bottom strand (bs) (Figure 1B) (14,15,18–20). This specific recognition of the folded bottom strand of *attC* sites allows the insertion of cassettes in the proper orientation so that they can be expressed by the P_C_ promoter (15,21). The recognition of this specific ssDNA substrate imposes some constraints for recombination reactions. Indeed, during the *attI* × *attC* reaction, an atypical Holliday junction (aHJ) is formed (Figure 1B), which cannot be resolved by the classical way but can be by a host-dependent replicative pathway (22). Several other host processes are implicated in cassette recombination, for instance, by influencing the proper folding of *attC* site (7,23). Host cells also control integrase and cassette expression (24–26). The most relevant regulatory network is the induction of integrase expression, for both Class 1 MI and *V. cholerae* SCI systems, in response to environmental stress through the SOS response (27,28). Such regulation allows the conditional reshuffling of cassettes, at a moment where the cells need to adapt to environmental changes. These examples show how integrons are intricate host-cell connected systems to maximize the potential benefit conveyed by this ‘adaptation on demand’ device (29).

Until now, the recombination processes occurring in integrons were mostly studied on MIs through assays developed and performed in *Escherichia coli* strains. Paradoxically, our knowledge of the SCI of the *V. cholerae* pathogenic strain remains predominantly descriptive more than 20 years after its discovery and despite its paradigmatic role in the field. Here, for the first time, we designed experimental assays to study cassette recruitment dynamics directly in the *V. cholerae* SCI. We delivered cassette substrates using conjugation, but also through natural transformation since *V. cholerae* is known to be naturally competent and to exchange DNA in this way. We measured significant cassette insertion rates mediated by the sole endogenous integrase. We confirmed that this integrase expression is due to the SOS activation probably triggered by the single-stranded cassette delivery during conjugation and transformation processes (24,30). Interestingly, cassettes are preferentially recruited into the *attIA* primary recombination site, directly downstream of the P_C_ promoter, ensuring their expression and consecutive testing for selective advantages they can confer.

By performing *in vivo* recombination assays, we showed that the RecA protein (RecA_Vch_) is critical for cassette recruitment at the *attIA* site of the *V. cholerae* SCI. The impact of RecA_Vch_ on cassette recombination is SOS-independent and seems specific to the *attIA* × *attC* reaction mediated by the integrase of *V. cholerae*, IntIA. Indeed, the RecA_Vch_ protein did not influence neither *attC* × *attC* recombination mediated by IntIA nor cassette recombination mediated by the MI Class 1 integrase, IntI1. Moreover, unlike that of IntI1, the *V. cholerae* SCI IntIA is not active in other bacterial hosts (e.g. in *E. coli*, even supplemented with RecA_Vch_). Altogether, these results suggest that, in contrast to MIs, some specific host factors can regulate cassette recombination in SCIs. Therefore, MIs might have been selected to be independent of such host factors. In addition to their association with transposons and conjugative plasmids, this evolutionary trait may explain the large MI dissemination among bacteria and the antibioresistance expansion.

## MATERIALS AND METHODS

### Media

*Escherichia coli* and *Vibrio cholerae* strains were cultivated at 37°C in Luria Bertani (LB) media. *V. cholerae* and *E. coli* strains containing a plasmid with a thermo-sensible origin of replication were grown at 30°C. Thymidine (Thy) and diaminopimelic acid (DAP) were supplemented, when necessary, to a final concentration of 0.3 mM. Glucose (Glu), l-arabinose (Ara) and isopropyl-β-d-thiogalactopyranoside (IPTG) were added respectively at final concentrations of 10, 2 mg/ml and 0.8 mM. To induce the P_tet_ promoter, anhydrotetracycline (aTc) was supplemented into the media to a final concentration of 1 μg/ml. In the case of *E. coli* strains, antibiotics were added at the following concentrations: carbenicillin (Carb), 100 μg/ml, chloramphenicol (Cm), 25 μg/ml, kanamycin (Km), 25 μg/ml and spectinomycin (Sp), 50 μg/ml. *V. cholerae* strains were cultivated with the same antibiotic concentrations except in the case of Cm and Sp, that were supplemented at a final concentration of 5 μg/ml and 100 μg/ml respectively. When *V. cholerae* strains were cultivated in presence of glucose, the later concentration of Sp was increased 2-fold (200 μg/ml).

### Bacterial strains, plasmids and primers

The different strains and plasmids that were used in this study are described in Supplementary Tables S1 and S2. All sequences of primers that were used are available in Supplementary Table S3.

We performed allelic exchange to construct N16961 *ΔrecA*, N16961 *ΔrecA ΔattIA*, N16961 *ΔrecA ΔattIA::attI1*, N16961 *hapR+ lexA*(ind-) and N16961 *hapR+ lexA*(ind-) *ΔrecA*. To this purpose, we used different variants of the pMP7 vector, respectively pB203, pK590, pK584 and p6780 (28). We followed the same protocols as previously described (31,32). Briefly, the suicide vector pMP7 contains a R6K origin of replication and its replication is then dependent on the presence of the Π protein in the host cell. The Π3813 cell, a *pir+* CcdB resistant *E. coli* strain (31), was used for cloning the different pMP7 plasmids. Once constructed, these vectors were transformed into the β3914 DAP auxotroph donor strain (31) in order to deliver by conjugation the pMP7 vector into the desired recipient strain. Homology regions corresponding to the genomic DNA from the recipient strain have been cloned in the different pMP7 vectors to allow the integration of the plasmid by homologous recombination. The only way for pMP7 vector to replicate within recipient strains is to then integrate into the host genome after a first crossover. After conjugation, integration of the entire pMP7 vector was then selected by plating cells on Cm plates lacking DAP. Next, cells were grown in presence of L-arabinose (0.2%) in order to express the CcdB toxin. The expression of this toxin allows the killing of cells in which the second crossover that leads to the excision of pMP7 backbone did not take place. This method allows us to obtain mutants of *V. cholerae* that are devoid of any antibiotic resistance marker. Note that for the deletion or replacement of the *attIA* site in N16961*ΔrecA* strains, we previously transformed this cell with pAM*::recA_Ec_* vector (pCY579 (33)). The expression of the RecA*_Ec_* protein allows allelic replacement to take place in N16961*ΔrecA* mutant. At the end of construction, strains were cultivated without Carb and IPTG and loss of the pAM*::recA_Ec_* plasmid was assessed. For ectopic complementation of the *recA* mutation, we inserted a copy of the *recA* gene into the *att*Tn7 site present on the chromosome of *E. coli* and *V. cholerae*. We used the same strategy as described in (34). The helper plasmid pMVM1 was transformed into both N16961 and MG1655 *recA* mutants. This vector has a thermo-sensitive origin of replication and carries a P_BAD_ promoter that triggers the expression of TnsABCD transposases. These transposases catalyze insertion into *att*Tn7 at high frequency. A second shuttle vector, pMP234, carries the IR sites that are recognized by the transposases and was modified for specific integration of the *recA* gene from *E. coli* or *V. cholerae*. The FRT-*aph*-FRT cassette was also added in between the IR, in order to select for transposition event. The pMP234 vector is a derivative of the pSW23T suicide vector, so its replication cannot take place into recipient cells that lack the Π protein. For integration, the pMP234 shuttle vector was delivered by conjugation into the recipient strains containing pMVM1. Transposition events were selected by plating conjugants on Km plates without DAP. These plates were incubated overnight at 42°C to get rid of the helper vector. The integration of P_LAC_-*recA*-FRT-*aph*-FRT fragment was assessed by testing UV sensitivity of the strains and by performing PCR and subsequent sequencing. After integration, the Flippase (Flp) expressing vector (pMP108, Carb^R^ (34)) was delivered by conjugation into the *V. cholerae* strain in order to excise the Km resistance cassette. This plasmid is easily lost when culturing *V. cholerae* strains without Carb. In the case of *E. coli*, we transformed the strains with the pCP20 Flp expressing vector (Carb^R^ (35)), which has a thermo-sensitive origin replication.

### Suicide conjugation assay

This assay has been previously described (14) and implemented (16) for the delivery of one specific strand of a recombination site into recipient strains that express the integrase. In this study, we used the suicide conjugative vector pSW23T (pD060) that allows the delivery of the bottom strand of the *attC_aadA7_* recombination site. An advantage of using this vector is its relatively small length (1817 bps) which enables to better mimicking the integron cassettes (7). This vector carries a RP4 origin of transfer and an *oriV*_R6Kγ_ origin of replication. It was previously transformed into a *pir+* donor strain, β2163, which contains the RP4 machinery of transfer. This later strain needs DAP to grow in rich medium, which allows its counter-selection after conjugation. The only possibility for pSW23T to replicate into the *pir-* recipient strains is thus to insert into the genome through a recombination reaction catalyzed by the IntI protein. Since the pSW23T vector contains a Cm^R^ cassette, recombination events can be selected with this marker. By plating in parallel conjugants on solid media with or without Cm, we are able to establish the frequency of a given recombination reaction. We adapted this protocol for the use of *V. cholerae* as the recipient strain, in which plasmids are more easily lost in absence of antibiotic selection than in *E. coli*. In this case, after an overnight culture, recipient cells were diluted (1:100) and grown in the presence of Sp and Ara (0.2%) respectively to maintain the pBAD43 vector and to allow the expression of the integrase. In the case of the *recA_Vch_* and *recA_Ec_* complemented strain, IPTG was also added in the media. The donor strain was grown in parallel in presence of DAP. When both donor and recipient cultures reach an OD_600 nm_ of 0.7–0.8, recipient *V. cholerae* strains were washed by centrifugation at 3000 rcf for 6 min and resuspension of the pellet in 1 ml of LB. 1 ml of each donor and recipient cultures were then mixed and centrifuged at 3000 rcf for 6 min. The obtained pellet was re-suspended in a droplet of LB and spread on a 0.45 μm filter placed on MH DAP, Ara plates and incubated at 37°C for 3 h. After incubation, the filter was re-suspended in 5ml of LB and this suspension was used to spread appropriate dilutions on MH, Cm, Sp, Glu and MH, Sp, Glu plates. After 2 days of incubation at 37°C, the recombination frequency was calculated as the ratio of Cm^R^ clones over the total number of recipient colonies that grew on MH, Sp, Glu plates. Note that in the case where we did not detect recombination events for one replicate, we calculate the recombination rates as the ratio of the mean of recombinant clones over the mean of total recipient clones obtained for the different replicates. In this case no error bars are represented on our graphs.

### Natural transformation assay

In this study, we used the same pSW23T vector (pD060) that was used in the suicide conjugation assay. *V. cholerae* natural transformation was previously described (36). Here, we adapted this protocol to assess integrase-mediated recombination frequency after natural transformation in competent strains of *V. cholerae*. As the natural *V. cholerae* N16961 strain contains a frameshift mutation in the *hapR* gene, which renders this strain non-transformable, we used the N16961 hapR+ strain, a genetically engineered N16961 variant with a repaired *hapR* gene. An overnight culture was used to inoculate 1:100 of bacteria in 5 ml of LB medium supplemented with Sp (50 μg/ml). The culture was grown at 30°C until they reached an OD_600nm_ of 0.5. One milliliter of cells was then centrifuged (2200 rcf, 10 min) and resuspended in 1 ml of M9 minimal medium supplemented with MgSO_4_ (32 mM) and CaCl_2_ (5 mM) and Sp (100 μg/ml). Tubes containing 50–80 mg of chitin (C9213; Sigma) were inoculated with 0.5 ml of washed cells and 0.5 ml of fresh M9 medium supplemented with MgSO4, CaCl_2_ and Sp, vortexed, and grown 48 h at 30°C with shaking. Cultures were then washed (2200 rcf, 10 min) and resuspended in an equal volume of fresh M9 medium supplemented with MgSO4, CaCl_2_, Sp and anhydrotetracycline (aTc) to induce integrase expression. The cultures were incubated again for 30 min at 30°C with shaking. Then, 2 μg of plasmid DNA (the pD060 vector) were added to the cultures and incubated for 36 h at 30°C with shaking. After incubation, bacteria were detached from chitin by vortexing vigorously for 30 s. The obtained bacteria suspension was used to spread appropriate dilutions on MH, Cm, Sp and MH, Sp plates. After 2 days of incubation at 37°C, the recombination frequency was calculated as the ratio of Cm^R^ clones over the total number of recipient colonies that grew on MH, Sp plates in the same manner as for suicide conjugation assay.

### Analysis of cassette insertion point localization

For each condition assay, at least 16 recombinant clones were isolated on appropriate plates and analyzed by PCR. For this, we performed different PCR reactions. In order to determine precisely if the pSW23T vector has been inserted into the *attIA* site of the SCI we used 5778 and SWend primers. These primers hybridize respectively in a sequence upstream of *attIA* in *V. cholerae* chromosome 2 or downstream of *attC_aadA7_* in the pSW23T vector. For *E. coli* and *V. cholerae* strains transformed with the pSU38Δ or on pBAD43 vectors that harbor recombination sites, SWbeg and MFD or 1704 and SWend primers respectively were used to amplify one junction of the co-integrate. Finally, to detect insertion of pSW23T in the genome of *V. cholerae*, either at secondary sites or into the VCR sites of the SCI, we performed random PCR amplification. For these, we performed a first randomized PCR reaction using degenerate 1863 and 2405 primers. The 2405 primer hybridizes upstream of the *attC* sites on pSW23T plasmids. Due to the presence of degenerate nucleotides in the 1863 primer, low hybridization temperatures were used, first, 30°C during 5 cycles and after, 40°C during 30 cycles. The obtained amplified DNA fragments were subjected to a second PCR reaction in order to enrich for PCR products corresponding to cassette insertion. For this purpose, we used primers 1865 and 1388. These primers hybridize respectively to the fixed part of the degenerated 1863 primer and upstream (but closer than 2405) of the *attC* sites on pSW23T plasmids. Recombination points were precisely determined by sequencing PCR products using primer 1366. The 1366 primer hybridizes upstream (but closer than 1388) of the *attC* sites on pSW23T plasmids. For each condition assay, at least three PCR reactions were purified and sequenced to confirm the insertion point.

### Recombination assay with unidirectional replicative substrate

This assay was previously described (23) and allows the determination of the recombination rates of a given recombination reaction when *attC* sites are carried by a replicative plasmid. The vectors that we used (p7523 or p7546, Cm^R^) replicate unidirectionally and the *attC_aadA7_* recombination sites they carry have been cloned so that the recombinogenic bottom strand is located either on leading or lagging strand template (‘lead’ or ‘lag’ orientation). *V. cholerae* strains were transformed with these unidirectional-replicative vectors and the IntIA expressing vector (p995, Carb^R^) or with the empty version of the plasmid (p979, Carb^R^). As p7523 and p7546 have both a thermo-sensitive origin of replication, the cells were cultivated overnight at 30°C. The next day, cultures were diluted (1:100) and incubated for 5h at 30°C in presence of Cm and Carb to maintain both pTSC29 and pBAD18 plasmid. In order for recombination to take place, L-arabinose was also added to the media at a concentration of 0.2% to allow the expression of the integrase. After 5h of incubation, appropriate dilutions of the cultures were plated in parallel on MH Cm, Carb, Glu and MH Carb, Glu plates and were incubated for two days at 42°C. Since pTSC29 vector cannot replicate at 42°C, the selection on Cm plates at this temperature allows the recovery of only the clones in which *attC_aadA7_* site have been recombined by IntIA. Recombination rates were calculated as for suicide conjugation assays, by considering the ratio of Cm^R^ clones over the total number of recipient clones that grew on MH Carb, Glu plates. In order to determine if cassette insertion occurs into the *attIA* site of the SCI of *V. cholerae*, we performed PCR reactions on at least eight random isolated clones using 5778 and 573 or 5778 and MFD primers (respectively used for ‘lead’ and ‘lag’ orientation of the bs of *attC_aadA7_* site on pTSC vector).

## RESULTS

### Horizontally transferred cassettes are efficiently inserted into the *attIA* site of the *Vibrio cholerae* SCI

**Figure 2.**
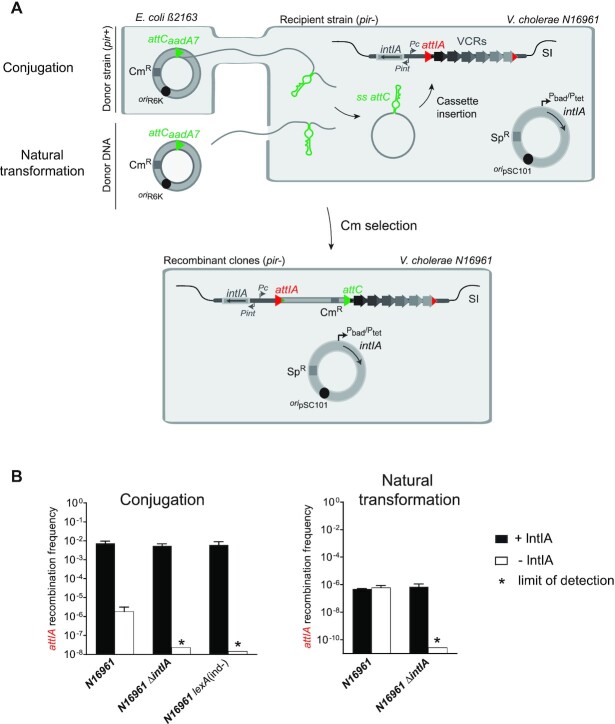
Cassette recruitment in *Vibrio cholerae* SCI during horizontal gene transfer. (**A**) Experimental setup of the cassette insertion assay. The pSW23T::*attC_aadA7_* suicide vector is delivered to N16961 *V. cholerae* recipient strains containing an integrase expressing vector or the sole endogenous integrase, and the SCI. The delivery occurs by two horizontal gene transfer processes: conjugation from the β2163 donor or natural transformation. As pSW23T cannot replicate in *V. cholerae* recipient strains, recombinant clones can be selected on appropriate Cm containing plates to evaluate the recombination frequency (see also Results and Materials and Methods). The *attC_aadA7_* site carried by the suicide vector is represented by a green triangle and the *attIA* site on the *V. cholerae* SCI by a red triangle. (**B**) Frequency of insertion of the pSW23T*::attC_aadA7_*suicide vector into the *attIA* site. The recombination frequencies were calculated in N16961 *V. cholerae* wt, *ΔintIA* and *lexA*(ind-) strains. Results correspond to recombination frequencies that were normalized after analysis of PCR reactions (Materials and Methods). +IntIA: recipient strains transformed with the pBAD43 integrase expressing vector; -IntIA: control strains transformed with the empty pBAD43 vector. * correspond to the limits of detection. Values represent the mean of at least three independent experiments and error bars correspond to average deviations from the mean.

Large SCIs such as the *V. cholerae* SCI constitute large reservoirs of functions for their host bacteria. In such massive integrons, several recombination sites can constitute potential targets for cassette insertion (*attIA* primary recombination site and/or the numerous VCR sites). In a previous study, recombination assays that were performed in *V. cholerae* aimed at evaluating cassette insertion in recombination sites carried on plasmids (16). Here, we attempted to visualize cassette insertion into the SCI of *V. cholerae*, using our classical conjugation assay (14) and developing a natural transformation assay. Interestingly, both assays reproduce the natural conditions in which the acquisition of cassettes could occur through horizontal gene transfer in *V. cholerae* (Figure 2A). The plasmids containing the donor *attC* site (pSW23T*::attC_aadA7_*) are delivered on a single-stranded form in a recipient *V. cholerae* strain containing a vector expressing the IntIA integrase. Once delivered, *attC*-containing plasmids cannot replicate and can therefore be assimilated to a non-replicative integron cassette. The unique way for these synthetic cassettes to be maintained is to be inserted in the *V. cholerae* host genome by a recombination between the cassette *attC* sites and the SCI *attI* or *attC* sites.

When we carried out this test, by delivering cassette by conjugation, we detected a significant level of insertion (7.5 × 10^–3^) of the pSW23T*::attC_aadA7_* vector using the N16961 *wt V. cholerae* strain (Figure 2B, +IntIA). We performed PCR reactions on some recombined clones (i.e. 24 clones) and demonstrated that, in all cases, the cassette insertion occurs at *attIA* sites from *V. cholerae* SCI platform (Supplementary Figure S1A). By sequencing some PCR products, we confirmed that insertion point was correctly localized in the 5′-AAC-3′ triplet.

We took advantage of *V. cholerae’*s natural competent state in the presence of chitin (37) to investigate cassette recombination in the context of another HGT mode. We adapted the natural transformation protocol to evaluate cassette insertion frequency in the *V. cholerae* SCI (Figure 2A). We obtained a recombination frequency of 4.8 × 10^–7^ when overexpressing the integrase (Figure 2B, +IntIA). We also performed PCR reactions on several recombined products (i.e. 32 clones) and demonstrated that cassette insertion occurs, for all tested clones, at *attIA* sites from *V. cholerae* SCI platform (Supplementary Figure S1B). These results show that, during conjugation or natural transformation, integron cassettes are efficiently released in the *V. cholerae* host cell and inserted in the SCI. Interestingly, all insertion events were detected in the integron platform *attIA* site in spite of the presence of about 180 *attC* sites.

### The endogenous integrase efficiently inserts cassettes into the *attIA* site of the *Vibrio cholerae* SCI

We also performed both assays in the presence of the sole endogenous SCI IntIA integrase. Interestingly, in this case, we detected a significant level of insertion of the pSW23T*::attC_aadA7_* vector using the N16961 *wt V. cholerae* strain (1.8 × 10^–6^ and 6.1 × 10^–7^ respectively for conjugation and transformation assays, Figure 2B, -IntIA). Here again, we performed PCR reactions on several recombined products and demonstrated that cassette insertion occurs, for all tested clones (i.e. respectively 16 and 48 clones), at *attIA* sites from the *V. cholerae* SCI platform (Supplementary Figure S1). This recombination activity is due to the expression of the endogenous *intIA* integrase gene since no recombination event was detected in the strain devoid of endogenous integrase (N16961 *ΔintIA*) for both assays (Figure 2B, -IntIA). We also demonstrated that the expression of endogenous integrase is dependant of the SOS system since no recombination event was detected below the detection limit of 1.5 × 10^–8^ in the N16961 *lexA*(ind-) strain in which the SOS response is not inducible. Indeed, in this strain, the SOS regulon genes are constitutively repressed because of the presence of the uncleavable LexA_A91D_ version of the LexA repressor (28). As a supplementary control, we also overexpressed the IntIA integrase in both *ΔintIA* and *lexA*(ind-) mutant strains and as expected we obtained a very high recombination frequency (respectively 5.4 × 10^–3^ and 6.0 × 10^–3^, Figure 2B, +IntIA) corresponding to insertion events at the *attIA* site from the *V. cholerae* SCI (96/96 and 24/24 clones were inserted in *attIA*, Supplementary Figure S1).

Altogether, these results show that, during conjugation or natural transformation, integron cassettes are efficiently delivered in the *V. cholerae* host cell and inserted at the *attIA* site from the *V. cholerae* SCI even in the presence of the sole endogenous integrase. The level of endogenous integrase expression, triggered by SOS response induction initiated by the single-stranded cassette entry during conjugation and natural transformation (24,30) seems sufficient to insert cassettes at a significant level in the *V. cholerae* SCI. Note that, when performing both conjugation and natural transformation, we also detected some *attIA* insertion events associated with shuffling of internal remote cassettes in first position (1 event on the 160 performed PCR for conjugation and 12 events on the 152 performed PCR for natural transformation, Supplementary Figure S1).

### RecA_Vch_ influences cassette insertion into the *attIA* site of the *Vibrio cholerae* SCI

Here, we precisely investigated the cassette recombination mechanism used by the SCI of *V. cholerae*. To define the network of intervening host factors, we tested the effect of the *Vibrio cholerae* RecA protein (RecA_Vch_). The RecA protein is functionally conserved among bacterial species (38) and in eukaryotic organisms (39). RecA is a critical enzyme for homologous recombination process, during which it binds ssDNA catalyzing the pairing with complementary regions of dsDNA and strand exchange reactions (40–42). Because of the capacity of the RecA protein to bind ssDNA, we tested its impact on SCI cassette recombination catalyzed by IntIA in *V. cholerae*.

**Figure 3.**
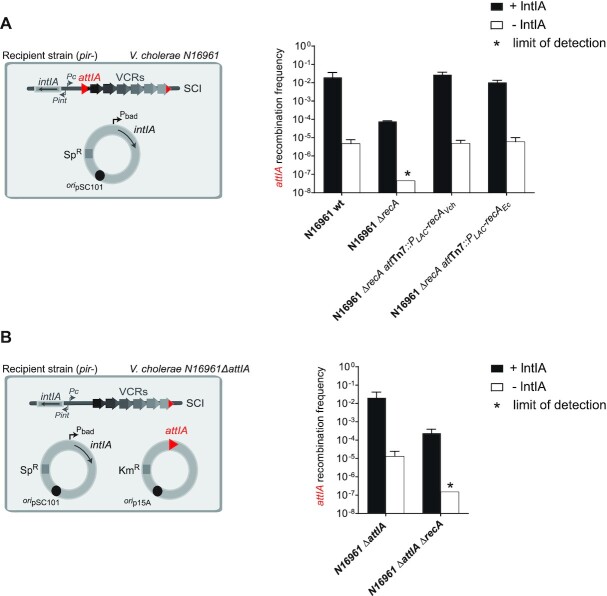
Effect of the RecA protein on *attIA* × *attC* recombination in *Vibrio cholerae* SCI. (**A**) Experimental setup and frequency of insertion of the pSW23T*::attC_aadA7_* suicide vector into the chromosomic *attIA* site. N16961 recipient strains transformed with the pBAD43 IntIA expressing vector were used (left panel). The recombination rates were calculated in N16961 *V. cholerae* wt and in the corresponding *recA* mutant (*ΔrecA*) and ectopic complemented (*ΔrecA-att_tn7_::*P_LAC_-*recA_Vch_* and *ΔrecA-att_tn7_::*P_LAC_-*recA_Ec_*) strains (right panel). (**B**) Experimental setup and frequency of insertion of the pSW23T*::attC_aadA7_*suicide vector into the *attIA* site located on plasmid. N16961 recipient strains transformed with both pBAD43 IntIA expressing vector and pSU38Δ*::attIA* vector were used (left panel). The recombination rates were calculated in N16961 *V. cholerae* wt and in the corresponding *recA* mutant strains (*ΔrecA*, right panel). For both (A) and (B), results correspond to recombination frequencies that were normalized after analysis of PCR reactions (Materials and Methods). +IntIA: recipient strains transformed with the pBAD43 integrase expressing vector; -IntIA: control strains transformed with the empty pBAD43 vector. * correspond to the limits of detection. Values represent the mean of at least three independent experiments and error bars correspond to average deviations from the mean.

Among the different conditions previously developed, we chose to use the optimal one, i.e. the suicide conjugation assay with overexpression of integrase (Figure 3A). Here again, we obtained a very high recombination rate (2.0 × 10^–2^) when using the *wt* parental strain as recipient strain. We observed a decrease of more than two orders of magnitude in the recombination rates (7.7 × 10^–5^) in the corresponding N16961 *ΔrecA* mutant strain (Figure 3A). When performing PCR analysis for each reaction, we confirmed that insertions occur in the *attIA* site (222/222 and 108/111, respectively for the *wt* and *ΔrecA* strains). These results mean that the RecA*_Vch_* protein favors insertion of cassettes in the *attIA* site of *V. cholerae* SCI. As expected, no recombination event was detected in the N16961 *ΔrecA* control strain that carries the empty pBAD43 vector (compare to the N16961 *wt* strain, Figure 3A), since the SOS response cannot be activated and induce the endogenous integrase expression in these cells. To confirm that the observed decrease in recombination rates is specifically due to the deletion of the *recA_Vch_* gene and not to polar effects, we constructed a strain in which the *recA_Vch_* deletion was ectopically complemented. For this, a copy of the *recA_Vch_* gene was inserted into the *att*Tn7 site located in the chromosome 1 of *V. cholerae* (34). This *recA_Vch_* ectopic complementation allows recovery of a recombination rate similar to the one of the N16961 *wt* strain (Figure 3A), meaning that the effect on *attIA* × *attC_aadA7_* recombination reaction, which we observed, is specific to the RecA*_Vch_* protein. Furthermore, we tested the ability of the RecA protein of *E. coli* (RecA*_Ec_*) to complement the *recA_Vch_* deletion. The *E. coli* and *V. cholerae* RecA proteins are highly similar, but concentrate their variations in their C-terminal part (Supplementary Figure S2). This C-terminal region is implicated in the modulation of RecA*_Ec_* activity notably by interacting with regulator proteins (43,44). We then tested if such variations could lead to host specific regulation that will impact cassette recombination. For this, we inserted a copy of the *recA* gene of *E. coli* into the *att*Tn*7* locus in *V. cholerae*. We observed that the complementation of the N16961 *ΔrecA* mutant with RecA*_Ec_* was as efficient as with the native RecA*_Vch_* of *V. cholerae* (Figure 3A). This is consistent with the high percentage of identity (almost 80%, Supplementary Figure S2) shared by both RecA proteins. Moreover, we observed that RecA*_Ec_* was also mediating SOS induction, leading to endogenous integrase expression in the control strain harboring the empty pBAD43 plasmid (Figure 3A). Both RecA proteins seem then to share enough identity so that the RecA*_Ec_* can replace RecA*_Vch_* for several functions, including its apparent role in cassette recombination in *V. cholerae*.

RecA is involved in the induction of the SOS response, thus its effect on cassette recombination could be indirect and may involve one or more proteins of the SOS regulon. Using the *lexA*(ind-) mutant, in which the SOS response is non-inducible, we did not observe any effect on recombination efficiency compared to the *wt* strain (Figure 2B) and, using the *lexA*(ind-) *ΔrecA* mutant, we still observe a large decrease of more than two orders of magnitude in the recombination rates (Supplementary Figure S3). When performing PCR analysis for each reaction, we confirmed that insertions occur in the *attIA* site (24/24 and 48/48, respectively for the *lexA*(ind-) and *lexA*(ind-) *ΔrecA* strains). These results allow us to demonstrate that the RecA*_Vch_* effect on IntIA mediated recombination is independent on the SOS regulon proteins. We also determined the role of the RecA*_Vch_* protein during cassette recombination in *attIA* when this site is located on a plasmid (Figure 3B). For this, we constructed a *V. cholerae* strain deleted for the resident *attIA* site (*V. cholerae* N16961 *ΔattIA*) and transformed with an *attIA*-containing plasmid. Once again, we obtained a decrease of two orders of magnitude (from 2.0 × 10^–2^ to 2.4 × 10^–4^, Figure 3B).

Altogether these results show that the RecA protein of *V. cholerae* favors the *attIA* × *attC* recombination mediated by IntIA, in a SOS-independent manner and independently of where *attIA* is localized.

### RecA_Vch_ does not influence *attC* × *attC* recombination in *Vibrio cholerae*

**Figure 4.**
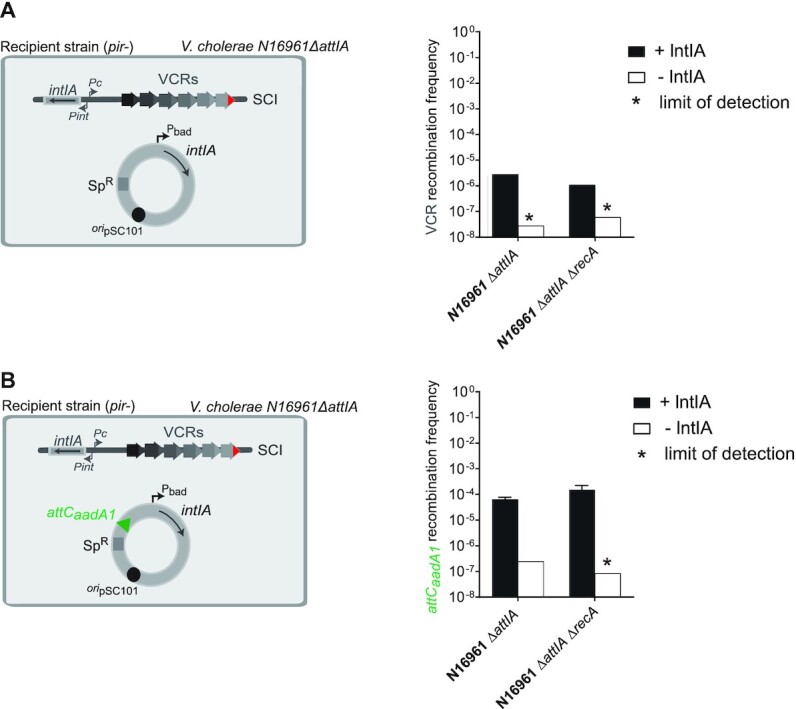
Effect of the RecA protein on *attC* × *attC* recombination in *Vibrio cholerae*. (**A**) Experimental setup and frequency of insertion of the pSW23T*::attC_aadA7_* suicide vector into VCR sites of the SCI. N16961 recipient strains deleted for the *attIA* site (*ΔattIA* strains) and transformed with the pBAD43 IntIA expressing vector were used (left panel). The recombination rates were calculated in N16961 *V. cholerae ΔattIA* and in the corresponding *recA* mutant strain (*ΔattIA ΔrecA*, right panel). (**B**) Experimental setup and frequency of insertion of the pSW23T::*attC_aadA7_*suicide vector into the *attC_aadA1_* site located on the pBAD43 plasmid. N16961 recipient strains deleted for the *attIA* site (*ΔattIA* strains) and transformed with the IntIA expressing and *attC_aadA1_* containing pBAD43 vector were used (left panel). The recombination rates were calculated in N16961 *V. cholerae ΔattIA* and the corresponding *recA* mutant strain (*ΔattIA ΔrecA*, right panel). For both (A) and (B), results correspond to recombination frequencies that were normalized after analysis of PCR reactions and sequencing (Materials and Methods). +IntIA: recipient strains transformed with the pBAD43 integrase expressing vector; -IntIA: control strains transformed with the empty pBAD43 vector. * correspond to the limits of detection. Values represent the mean of at least three independent experiments and error bars correspond to average deviations from the mean.

During both *attC* × *attC* and *attI* × *attC* reactions, the proper folding of *attC* site is essential for binding integrase monomers. Thus one could imagine that the effect seen for RecA here could be linked to its ssDNA binding function. In this case, we expect that it should impact both *attI* × *attC* and *attC* × *attC* reactions that are catalyzed by IntIA. To test this, we used different assays that allow assessment of cassette insertion frequencies directly into the VCR sites of the SCI or in *attC* sites carried on plasmids (Figure 4). To observe insertion events into the VCR sites of the SCI, we used the *V. cholerae* mutant strain deleted for the resident *attIA* site (*V. cholerae* N16961 *ΔattIA*, see just above). Here, we did not observe any differences in the recombination rates obtained with *ΔattIA* and *ΔattIA ΔrecA_Vch_* mutant strains. To confirm the cassette insertion in VCRs, we performed random PCR reactions (Materials and methods). As the recombination rates into VCR sites is similar between both strains (Figure 4A), this means that the RecA*_Vch_* protein does not affect the *attC* × *attC* recombination. Note that, in our experiment, the natural expression of the endogenous integrase is not high enough to mediate cassette insertion events in VCR sites at a detectable level. Interestingly, overexpressing the integrase enables to access to these rare recombination events and therefore to study the mechanism of integron recombination in more details.

To confirm this absence of RecA*_Vch_* effect on *attC* × *attC* recombination, we perform a second conjugation assay in *V. cholerae* N16961 *ΔattIA* strains transformed with a plasmid carrying the *attC_aadA1_* site (from the *aadA1* gene cassette found in MIs). We obtained a significant rate of recombination for both *ΔattIA* and *ΔattIA ΔrecA_Vch_* strains and we did not observe any impact of the RecA*_Vch_* protein on *attC_aadA1_* × *attC_aadA7_* reaction catalyzed by IntIA in *V. cholerae* (Figure 4B). Together, these results, with those presented in the previous paragraph, indicate that the RecA*_Vch_* protein seems to favor only *attIA* × *attC* reaction and has no influence on insertion reactions into *attC* sites, whether they are present on the chromosome (VCR) or on a plasmid (MI *attC* sites).

### RecA_Vch_ does not influence *attI1* × *attC* recombination mediated by IntI1 in *Vibrio cholerae*

**Figure 5.**
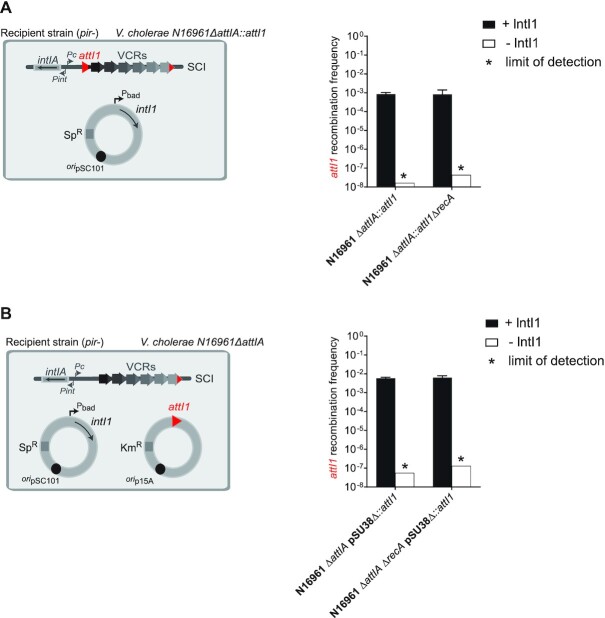
Effect of the RecA protein on *attI1* × *attC* recombination mediated by IntI1 in *Vibrio cholerae*. (**A**) Experimental setup and frequency of insertion of the pSW23T*::attC_aadA7_*suicide vector into the *attI1* site located in the SCI platform. N16961 recipient strains with an *attI1* site in place of the *attIA* site (*ΔattIA::attI1* strains) were transformed with the pBAD43 IntI1 expressing vector (left panel). The recombination rates were calculated in N16961 *V. cholerae ΔattIA::attI1* and the corresponding *recA* mutant strain (*ΔattIA::attI1 ΔrecA*, right panel). (**B**) Experimental setup and frequency of insertion of the pSW23T*::attC_aadA7_*suicide vector into the *attI1* site located on a plasmid. N16961 recipient strains deleted for the *attIA* site (*ΔattIA::attI1* strain) and transformed with both pBAD43 IntI1 expressing vector and pSU38Δ*::attI1* vector were used (left panel). The recombination rates were calculated in *V. cholerae ΔattIA::attI1* and the corresponding *recA* mutant strain (*ΔattIA::attI1 ΔrecA*, right panel). For both (A) and (B), results correspond to recombination frequencies that were normalized after analysis of PCR reactions (Materials and Methods). +IntIA: recipient strains transformed with the pBAD43 integrase expressing vector; -IntIA: control strains transformed with the empty pBAD43 vector. * correspond to the limits of detection. Values represent the mean of at least three independent experiments and error bars correspond to average deviations from the mean.

The previously demonstrated influence of the RecA*_Vch_* protein on cassette recombination was striking since it has been previously shown that the RecA*_Ec_* protein does not influence the recombination reactions catalyzed by IntI1 in *E. coli* (45). To determine if the role of the RecA*_Vch_* protein is specifically linked to the *V. cholerae* SCI system, we used two different recombination tests to establish, in *V. cholerae*, the activity of the integrase IntI1. First, we performed the suicide conjugation assays in a *V. cholerae* strain where the *attIA* site from the SCI platform was replaced by the *attI1* site (the *attI* site of the IntI1 integrase). Secondly, we performed this assay in *V. cholerae* strains deleted for the *attIA* site (*V. cholerae* N16961 *ΔattIA*) and transformed with an *attI1*-carrying plasmid. We observed, using these two assays, that the frequencies of *attI1* × *attC* reaction catalyzed by IntI1 were similar for both *ΔattIA* and *ΔattIA ΔrecA* recipient strains (Figure 5A and B). Therefore, we demonstrated that, in *V. cholerae*, the *attI1* × *attC* reaction catalyzed by IntI1 is not influenced by RecA*_Vch_*. This suggests that the role of RecA*_Vch_* on the *attI* × *attC* reaction is specific to IntIA.

### Effect of RecA on cassette recombination in *Escherichia coli*

**Figure 6.**
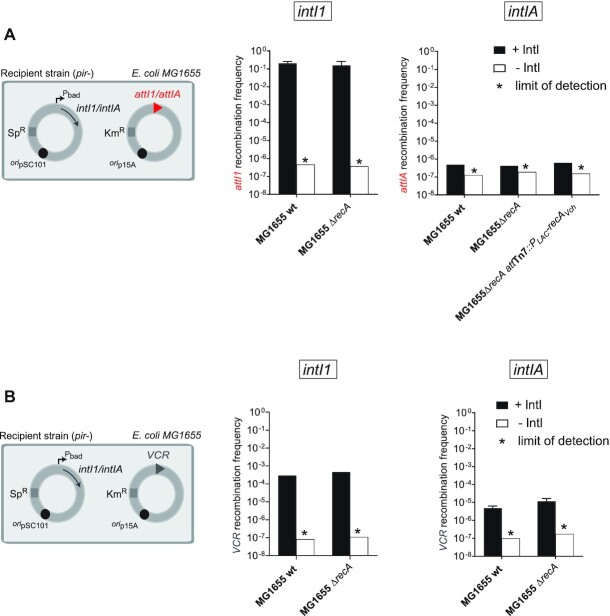
Effect of the RecA protein on recombination reactions catalysed by IntI1 and IntIA in *Escherichia coli*. (**A**) Experimental setup and frequency of insertion of the pSW23T*::attC_aadA7_*suicide vector into the *att* sites (*attI1* or *attIA*) located on a plasmid. MG1655 recipient strains transformed with both pBAD43 IntI expressing vector (IntI1 or IntIA) and the pSU38Δ*::attI* vector (*attI1* or *attIA*) were used (left panel). The recombination rates were calculated in MG1655 and in the corresponding *recA* mutant (*ΔrecA*) and ectopic complemented (*ΔrecA-att_tn7_::*P_LAC_-*recA_Vch_*) strains (right panels). (**B**) Experimental setup and frequency of insertion of the pSW23T*::attC_aadA7_*suicide vector into the VCR_VCA0441_ site located on a plasmid. MG1655 recipient strains transformed with both pBAD43 IntI expressing vector (IntI1 or IntIA) and the pSU38Δ::VCR_VCA0441_ vector were used (left panel). The recombination rates were calculated in MG1655 and in the corresponding *recA* mutant strain (*ΔrecA*, right panels). For both (A) and (B), results correspond to recombination frequencies that were normalized after analysis of PCR reactions (Materials and Methods). +IntI: recipient strains transformed with the pBAD43 integrase expressing vector; -IntI: control strains transformed with the empty pBAD43 vector. * correspond to the limits of detection. Values represent the mean of at least three independent experiments and error bars correspond to average deviations from the mean.

As already mentioned, the impact of the RecA protein on cassette recombination had already been tested in *E. coli* (45). In this previous study, a setup based on the reconstitution of a functional *dapA* gene after cassette excision through an intramolecular reaction was used. In this case, only the efficiency of reactions catalyzed by the IntI1 integrase was studied (45). Here, we extended this by assessing the efficiency of either *attI* × *attC* or *attC* × *attC* intermolecular reactions and tested the effect of *recA* deletion on reactions catalyzed by both IntI1 and IntIA integrases in *E. coli*. We also used another *attC* site, VCR_VCA0441_ (45). We still observed the RecA-independence of IntI1 for both *attI1* × *attC_aadA7_* and VCR × *attC_aadA7_* reactions (Figure 6A and B, left panels). Indeed, respective recombination frequencies were identical in MG1655 *wt* and *ΔrecA* recipient strains. For the reaction catalyzed by IntIA, we observed that, in *E. coli*, the VCR × *attC_aadA7_* reaction was not affected by the deletion of the *recA* gene (Figure 6B, right panel). In accordance with the study of Biskri and coll. (16), we found that IntIA*_Vch_* was not able to efficiently catalyze the *attIA* × *attC_aadA7_* reaction in *E. coli* in the *wt* strain (Figure 6A, right panel). As proposed earlier, if IntIA recombines much less efficiently in *E. coli* this may reflect the absence, or a too large divergence, of at least one host factor required for this reaction. Thus, in order to determine if RecA could correspond to one of these divergent factors, we tested if RecA*_Vch_* could rescue the lack of *attIA* × *attC* recombination in *E. coli*. As performed previously in *V. cholerae*, we inserted the *recA_Vch_* gene into the unique *att*Tn*7* site of *E. coli*. However, we found that the expression of RecA*_Vch_* do not restore the recombination capacity of IntIA for the *attIA* × *attC_aadA7_* reaction in *E. coli*. Thus, it shows that the RecA protein is not the unique factor whose divergence hampers the *attIA* × *attC_aadA7_* reaction catalyzed by IntIA to take place in *E. coli*.

### RecA_Vch_ influences *attIA* cassette insertion no matter how the *attC* site is delivered

**Figure 7.**
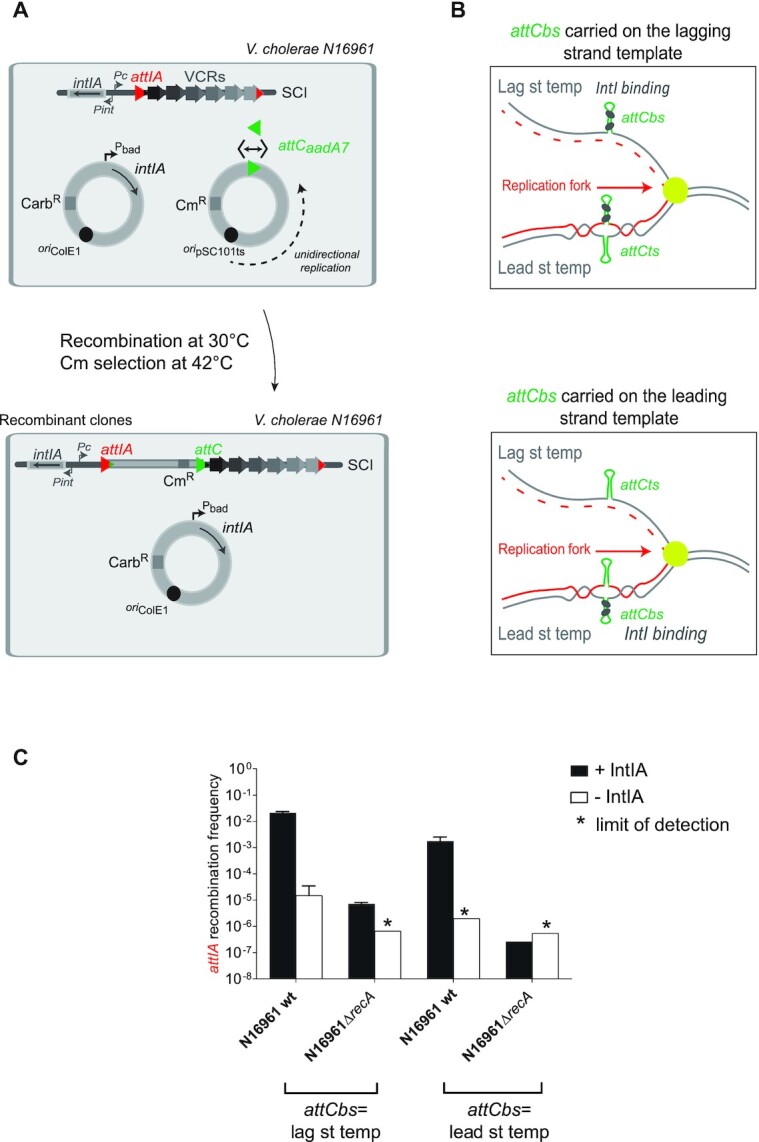
Effect of the RecA protein on *attC* × *attI* reaction when *attC* sites are carried by a replicative vector in *Vibrio cholerae*. (**A**) Experimental setup of the replicative assay. *V. cholerae* N16961 strains transformed with both pBAD18 IntIA expressing vector and the unidirectional-replicative vector, pTSC29::*attC_aadA7_* were used. *attCaadA7* sites (green triangles) were cloned in both orientations (double arrow) in the pTSC29 vector. Since pTSC29 has a thermosensitive origin of replication, the recombination reaction is performed at 30°C and, to evaluate the recombination frequency, recombinant clones selection was performed on Cm containing plates at 42°C (see also Results and Materials and methods). The *attIA* site on the *V. cholerae* SCI is represented by a red triangle. (**B**) Folding of *attC_aadA7_* site on a replicative vector. Replicated and template strands are coloured in red and grey respectively and grey circles represent IntIA monomers. bs: bottom strand; ts: top strand; Lag st temp: Lagging strand template; Lead st temp: Leading strand template. (**C**) Frequency of insertion of the pTSC29::*attC_aadA7_*unidirectional-replicative vector into the *attIA* site. The recombination rates were calculated in N16961 *V. cholerae* wt and in the corresponding *recA* mutant strains (*ΔrecA*). Under plots, the orientation of *attC_aadA7_* bs on lagging or leading strand template (lag st temp or lead st temp) are indicated. Results correspond to recombination frequencies that were normalized after analysis of PCR reactions (Materials and Methods). +IntI: recipient strains transformed with the pBAD18 integrase expressing vector; -IntI: control strains transformed with the empty pBAD18 vector. * correspond to the limits of detection. Values represent the mean of at least three independent experiments and error bars correspond to average deviations from the mean.

In all previously presented assays, we used horizontal gene transfer mechanisms as a means for delivering the single-stranded recombinogenic bottom strand of the *attC* site on non-replicative substrate mimicking integron cassettes. To determine if RecA still favors cassette insertion in the case where *attC* sites are folded from double-stranded molecules, we performed a recombination assay that relies on the use of replicative vectors (pTSC29 derivatives). These vectors replicate unidirectionally and the *attC_aadA7_* site that they carry was cloned in two orientations, so that the bottom strand of the *attC* site is either present on the lagging or leading strand template (Figure 7A). When the bs of the *attC* site is located on the lagging strand template, in which large region of ssDNA are available during replication (i.e., between Okazaki fragments), bs *attC* site folding from ssDNA is favored (Figure 7B). When the bs of the *attC* site is located on the leading strand template, bs *attC* site folding can only occur from dsDNA by cruciform structure extrusion (23) (Figure 7B). Since the pTSC29 vectors have a thermo-sensitive origin of replication, the selection of recombinant Cm^R^ clones at 42°C allows us to evaluate the efficiency of recombination events. When we performed this replicative assay in the *V. cholerae* N16961 *wt* strain, we observed a decrease of about one order of magnitude when the *attC_aadA7_* bs is present on the leading strand template (Figure 7C). Such differences in the recombination frequency between the two orientations of the *attC_aadA7_* site were previously observed when the same recombination assays were carried out in *E. coli* with the integrase IntI1 (23). In the N16961 *ΔrecA* mutant, we still observed a large decrease in recombination rates for both *attC_aadA7_* site orientations (Figure 7C). These results demonstrate that RecA*_Vch_* is involved in the mechanism of recombination during *attIA* × *attC* reaction whatever the manner that the *attC* site is delivered (i.e. from ssDNA or dsDNA).

## DISCUSSION

### Incoming integron cassettes are efficiently inserted in the *attIA* site of the *Vibrio cholerae* integron

Exchange of integron gene cassettes between bacterial species (46) and gene cassette shuffling in the *V. cholerae* SCI (30) have been detected previously but at very low frequencies and have only been linked to homologous recombination events. Here, for the first time, we experimentally succeeded to visualize cassette recruitment events in the SCI of the *Vibrio cholerae* pathogenic strain, catalyzed by the sole endogenous integron integrase. We used both conjugation and natural transformation processes to deliver the integron cassettes through horizontal gene transfer in *V. cholerae* strains. Up to now, we always used our classical conjugation assay to deliver cassettes in recipient strains and study integron recombination. However, this assay does not perfectly mimic the integron system since cassettes are not themselves conjugative elements and have to be excised from the conjugative plasmid before their insertions. This is why we attempted here to develop for the first time another assay involving natural transformation, which is thought to be an important road for cassette recruitment in *V. cholerae*.

**Figure 8.**
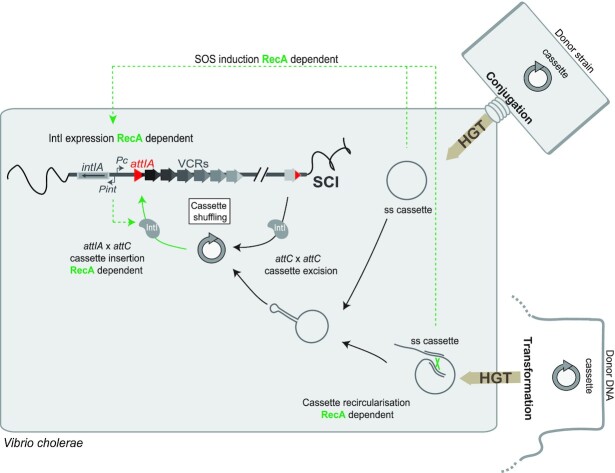
Snap shot of SCI recombination during horizontal gene transfer in *Vibrio cholerae*. The SCI activity is represented and its connections with bacterial physiology. The steps, which involve the RecA protein, are indicated in green. HGT: horizontal gene transfer; ss: single-stranded DNA; VCR: *Vibrio cholerae* Repeat sequences; SCI: sedentary chromosomal integron.

When delivering cassettes by conjugation assay, we detected a high rate of cassette insertion (∼10^–2^) in conditions where IntIA is overexpressed. However, we were also able to detect a significant level of cassette insertion (>10^–6^) catalyzed by expression of the sole endogenous integrase. We also detected cassette insertion events catalyzed by the endogenous integrase by delivering cassettes during natural transformation. The entry of single-stranded cassettes by natural gene transfer processes activates the SOS response sufficiently to express the endogenous integrase at levels allowing the proper insertion of these incoming cassettes. We therefore reproduce the whole natural integron pathway in which single-stranded integron cassettes entry, mediated by HGT processes, activates their proper insertion in the integron platform (Figure 8).

Interestingly, by analyzing the insertion events, we demonstrated that despite the presence of about 180 VCR sites in the large array of cassettes, the *attIA* site constitutes the major recruitment site for cassette insertion in the *V. cholerae* SCI. It has been demonstrated that expression levels are maximal for these first cassettes in the array, and gradually decrease for those following (47,48). Nonetheless, in the large cassette arrays such as those of SCIs, the cassette expression level can vary along of the array depending on the nature of the cassettes (49,50). First, even though these are a minority, cassettes can carry their own promoters and therefore be expressed whatever their position in the array. Second, cassettes can have a RBS site ensuring a better translation (51). Third, for cassettes devoid of RBS, the presence of translated ORF just upstream increases their translation rates favoring the destabilization of the *attC* site structure on the mRNA transcript and ensuring the ribosome progression (9). Nevertheless, these observed preferential insertions of incoming cassettes in the first position, i.e. in the P_C_ vicinity, ensure their efficient expression whatever their nature. This means that, even if the frequency of cassette insertion seems low when mediated by the endogenous integrase (∼10^–6^), these events can be selected and evolutionarily fixed if cassette expression leads to an advantageous new function. Thus, the knowledge of the precise cassette functions in SCI would allow us to trace back the environmental conditions of cell growth and the evolutionary history of SCI-containing cells.

### RecA_Vch_ is critical for cassette insertion in the *attIA* site of the *Vibrio cholerae* SCI

Previous studies have shown how integrons are intimately connected with their hosts. For instance, the folding of single-stranded *attC* substrates depends on several cellular processes including conjugation, replication or DNA topology (7,23). *attC* site folding can also be modulated by the binding of the SSB host protein, which, by hampering *attC* hairpin folding *in vivo* in the absence of IntI, would play an important role in maintaining *attC* integrity and thus its recombinogenic functionality (45). It has also been shown that the pathway that allows the resolution of aHJ formed during the *attI* × *attC* reaction is linked to a host-replicative process (22). Moreover, integrase expression is also extensively regulated by environmental stresses, thus connecting cassette recombination to the host-cell environment. Both integrase and cassette expressions, ensured respectively by P_int_ and P_C_, are regulated by catabolite repression in *V. cholerae* SCI (24,26). Expression of the MI class 1 integrase is in part regulated by the stringent response in biofilms (25). The most relevant example among these regulatory pathways is undoubtedly the induction of integrase expression (of class 1 MI and *V. cholerae* SCI) during the SOS response. Such regulation is ensured by the binding of the LexA repressor in the promoter region of P_int_ (27,28). Integrase expression, similar to all genes of the SOS regulon (more than 40 SOS genes) (50), is triggered by the autocatalytic cleavage of LexA, a process induced by the binding of RecA on single-stranded DNA, constituting a nucleofilament. The SOS response is a global response to DNA damage ensuring a state of high-activity DNA repair.

In this study, we investigated the role of the RecA protein at another regulatory level of the integron system, cassette recombination. We choose to study this protein for two reasons. First, because of its ssDNA binding properties, since we know that cassette recombination involves one of the recombination sites, the *attC* site, in a ssDNA form. Secondly, because efficient cassette recombination could be dependent on one or several proteins belonging to the SOS regulon and therefore whose expression is induced in presence of RecA. We found that the RecA*_Vch_* protein is critical for the *attIA* × *attC* reaction in the SCI of *V. cholerae* and that the RecA effect is independent on SOS regulon proteins. On the contrary, when we tested the effect of RecA*_Vch_* on cassette insertions in VCR sites of the SCI, we found that these *attC* × VCR reactions were independent of the RecA*_Vch_* protein. We also confirmed the absence of the RecA effect on *attC* × *attC* recombination by testing cassette insertion in an *attC_aadA1_* site-carrying plasmid. Interestingly, these results show that the RecA effect depends on the nature of *att* sites (*attI* or *attC*), which are recombined. Altogether, our results indicate that the RecA protein is a host factor specifically involved in the cassette recruitment in the *attIA* site of the *V. cholerae* SCI during environmental stress conditions. Finally, the RecA protein acts at several steps during the integron recombination process (Figure 8). First, as described above, in the presence of antibiotics or when single-stranded cassettes are released in the cell by HGT, RecA favors the expression of the SOS-dependent integrase gene (28,52). Secondly, RecA ensures the cassette recruitment in the SCI of *V. cholerae*. Since the *recA* gene itself also belongs to the *V. cholerae* SOS regulon (50), its expression is probably up-regulated as in *E. coli* (ten-fold within minutes (53,54)). This release of the RecA protein would allow RecA to be available to trigger its biological functions, among which its effect on cassette recruitment at *attIA* SCI (Figure 8).

### Model of RecA_Vch_ mechanism of action

RecA nucleofilament indirectly activates expression of a large number of genes during the SOS response (50). The mechanism of action of RecA*_Vch_* on integron recombination could thus be the consequence of an indirect effect. Using a *V. cholerae* mutant strain *lexA*(ind-) in which the SOS response is constitutively repressed, we showed that SOS response abrogation did not affect recombination rates and therefore that the observed RecA*_Vch_* effect on *attIA* × *attC* recombination does not involve any other protein belonging to the SOS regulon. Besides, we know that the *E. coli* RecA protein differs from the *V. cholerae* RecA protein in the extreme C-terminal part and that this part is known to modulate interactions with regulatory proteins (Supplementary Figure S2) (Cox, 2007b). We decided to study the effect of the RecA*_Ec_* protein in *attIA* × *attC* recombination mediated by IntIA. We performed complementation of the *V. cholerae ΔrecA* mutant by the *recA_Ec_* gene. We observed that the RecA*_Ec_* protein fully restores the recombination activity, meaning that both RecA proteins may ensure cassette insertion in the *attIA* site.

Contrary to the *attC* × *attIA* reaction, we did not observe any influence of RecA*_Vch_* on *attC* × *attC* reaction mediated by IntIA in *V. cholerae*, while both reactions require a ss *attC* site on a double-stranded DNA molecule, we still observed an important effect of the RecA*_Vch_* protein. Then, in whatever way *attC* sites are delivered, i.e. single-stranded (conjugation and replicative assays) or double-stranded (replicative assay), we observed an important effect of the RecA*_Vch_* protein in *attIA* × *attC* recombination. Altogether, these results show that the observed RecA*_Vch_* effect is not linked to the regulation of *attC* site folding. We therefore suppose that the *attIA* × *attC* synaptic complex formed during the recombination could impede the aHJ resolution by blocking replication fork progression. The effect of RecA would be to bind the ss *attC* sites and help to maintain the *attC* site in a non-folded single-stranded form destabilizing the synaptic complex and favoring the replicative resolution step. Note that we did not observe this RecA*_Vch_* effect on *attC* × *attC* recombination suggesting a differential configuration between both *attC* × *attC* and *attIA* × *attC* complexes. Indeed, synapses formed during both reactions are known to be different since involving respectively two flexible bs *attC* sites versus a flexible bs *attC* and a stiffer ds *attI* (55). Such differences would explain that the synapse architecture for the *attIA* × *attC* reaction versus *attC* × *attC* may potentially require the assistance of additional accessory proteins. Furthermore, the *attC* × *attC* reaction can be catalyzed by IntIA in *E. coli* while, productive *attIA* × *attC* reactions are almost undetectable in this host (16).

### Host factor recruitment differs between mobile and sedentary chromosomal Integrons

Biskri *et al.* previously observed the integrase of the SCI of *V. cholerae* recombines 2000-fold less efficiently during an *attIA* × *attC* reaction when expressed in a heterologous host such as *E. coli* (16). This observation suggests that IntIA requires host factors that are absent or too divergent in *E. coli* to carry out this reaction (16). Here, we confirmed this result and failed at recovering *attIA* × *attC* recombination by expressing RecA*_Vch_* in *E. coli*, suggesting that the RecA*_Vch_* protein is not the missing factor (or not the single one) in *E. coli* impeding this reaction to efficiently occur. This is not surprising since, even though they show some slight variations in the C terminal part, RecA*_Ec_* and RecA*_Vch_* present a very high level of identity (80%, Supplementary Figure S2). In addition, this is in accordance with the fact that RecA*_Ec_* was able to functionally complement the absence of RecA*_Vch_* to achieve this reaction in *V. cholerae* (Figure 3A). Therefore, we hypothesize that other specific *V. cholerae* host factors are missing in *E. coli*.

In contrast, RecA is not involved in any reactions mediated by IntI1 (neither in *E. coli* or *V. cholerae*) and all these reactions are efficient in *V. cholerae*. Therefore, while SCI cassette recombination activity seems restricted to a given host, MI recombination seems efficient in different species suggesting a host factor recruitment more stringent for SCIs compared to MIs. This is consistent with the observed widespread MIs dissemination among bacterial species. The evolutionary success of class 1 MIs clearly reflects the fact that they are functional in a wide range of bacterial hosts. This could result from a co-evolution between the integrase IntI1 and its *attI1* site to ensure that cassette recombination takes place in absence of specific accessory proteins. Both IntI and *attI* level of divergence, 45% between IntIA and IntI1 (55) and the lack of identity between *attIA* and *attI1* sites, likely reflect their functional differences. Indeed, the *attI1* site harbors supplementary IntI1 binding sites called ‘direct repeats’ (DRs, Supplementary Figure S4), that constitute accessory sequences to which the integrase is able to bind (56). These DRs favor *attI1* × *attC* reactions (57). However, such sequences are absent from the majority of *attI* sites (58) and, for instance, the *attIA* site of the *V. cholerae* SCI does not harbor any DRs (Supplementary Figure S4). Upon mobilization of integrons, *attI* sites may have evolved and selected such motifs (e.g. DRs), to replace trans-acting host factors necessary for the *attI1* × *attC* reactions. For instance, DRs of *attI1* site acting as topological filters (57) could therefore replace host factors that regulate supercoiling.

### Natural transformation represents an efficient pathway for SCI cassette recruitment

HGT contributes to the emergence of pathogens and the spread of virulence factors, and also enables many disease-causing bacteria to rapidly evolve in response to environmental pressures such as antibiotic use (59–65). Among HGT processes, natural transformation is able to mediate the absorption and exchange of free DNA when sufficient homology is present between the incoming DNA and the bacterial genome (66,67). Here, we demonstrated that acquisition of material during natural transformation can be also mediated by homology independent DNA mechanisms, notably by exchanging and recruiting gene cassettes inside integrons in an integrase dependent way. We used the causative agent of the diarrheal disease cholera, *Vibrio cholerae*, responsible for seven major pandemics since 1817, the latter of which being still ongoing (68). Cassette recruitments occur directly in the *attIA* site of the SCI contained in *V. cholerae* where they are expressed and selected if providing an advantage to the *V. cholerae* strain. The natural bacterial competence has already been demonstrated for at least 80 species of bacteria but remains little explored and is probably underestimated (69,70). Notably, natural transformation is largely widespread among *Vibrionaceae*, where SCIs are mainly found and constitute very plastic regions (71,72). Here, we validated a new pathway for integron cassette recruitment in SCIs triggered by natural transformation probably involved in adaptation and making integrons important motors of evolution in *Vibrionaceae* species.

## Supplementary Material

gkab412_Supplemental_File
