# Supplementary material for: Cassette recruitment in the chromosomal Integron of Vibrio cholerae
Source: Nucleic Acids Res. 2021 May 28;49(10):5654–70. doi: 10.1093/nar/gkab412 (PMC8191803; doi:10.1093/nar/gkab412)
Supplement: gkab412_Supplemental_File [file gkab412_supplemental_file.pdf]

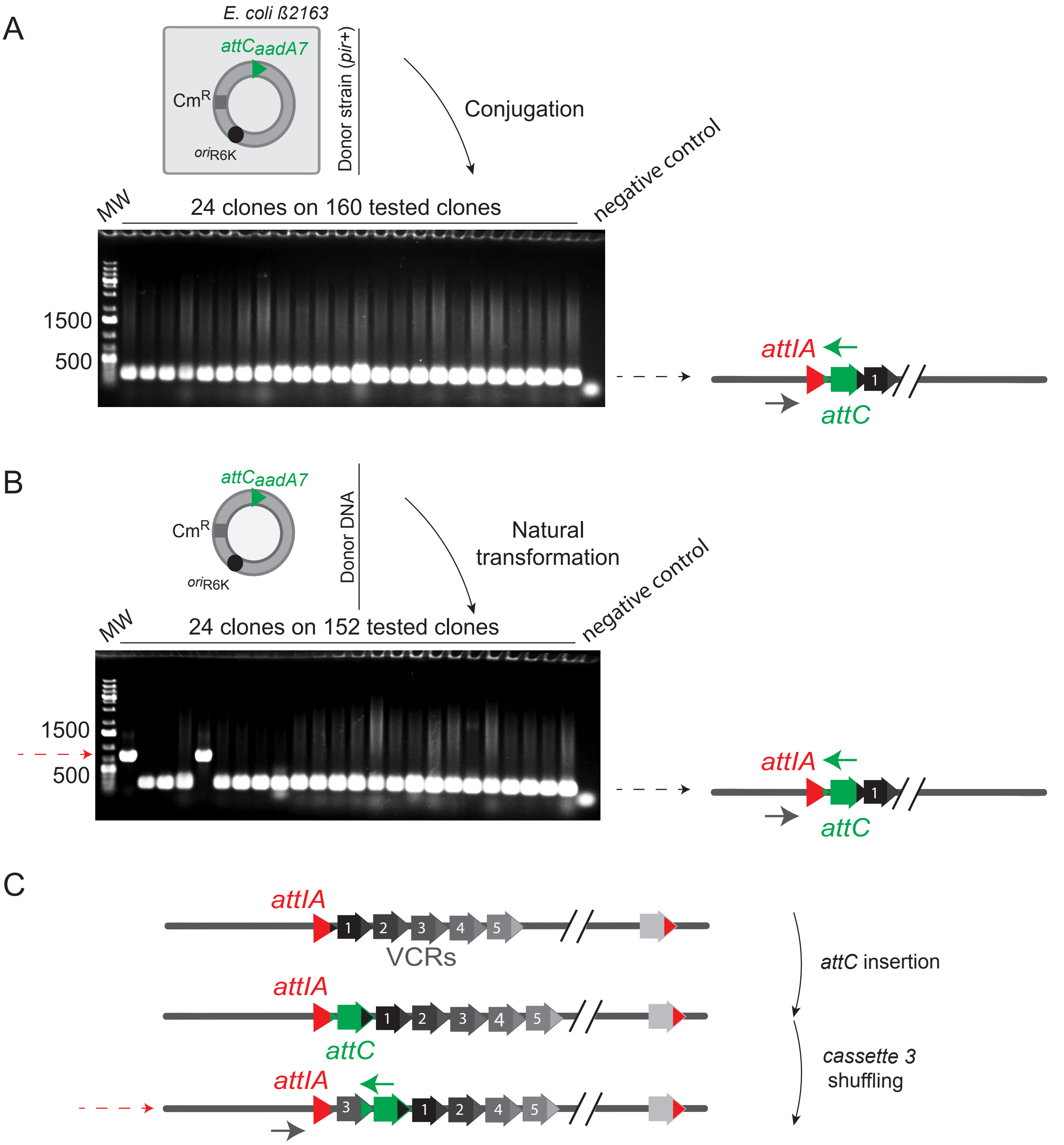

Figure S1: **Analysis of recombined clones**

A and B. Electrophoresis analysis of PCR products (24 represented on a total of 160) after conjugation (A) and of PCR products (24 represented on a total of 152) after natural transformation (B). Reactional products and used primers for PCR (grey and green arrows) are shown. The *attC_{aadA7}* site carried by the suicide vector is represented by a green triangle. MW: Molecular Weight Marker DNA (C) Schemes showing the successing events (*attC_{aadA7}* cassette insertion followed by cassette 3 shuffling) leading to reactional products showed by the dashed red arrow in B .
The *attIA* site on the *V. cholerae* SCI is represented by a red triangle and VCR sites by arrows.

```
RecAEc      1  MAIDENKQKALAAALGQIEKQFGKGSIMRLGEDRSMDVETISTGSLSLDIALGAGGLPMG  60
                 +DENKQKALAAALGQIEKQFGKGSIMRLG++R+MDVETISTGSLSLDIALGAGGLPMG
RecAVch     1  --MDENKQKALAAALGQIEKQFGKGSIMRLGDNRAMDVETISTGSLSLDIALGAGGLPMG  58

RecAEc     61  RIVEIYGPESSGKTTLTLQVIAAAQREGKTCAFIDAEHALDPIYARKLGVDIDNLLCSQP  120
               RIVEI+GPESSGKTTLTL++IAAAQREGKTCAFIDAEHALDP+YA+KLGV+ID LL SQP
RecAVch    59  RIVEIFGPESSGKTTLTLELIAAAQREGKTCAFIDAEHALDPVYAKKLGVNIDELLVSQP  118

RecAEc    121  DTGEQALEICDALARSGAVDVIVVDSVAALTPKAEIEGEIGDSHMGLAARMMSQAMRKLA  180
               DTGEQALEICDALARSGAVDVIVVDSVAALTPKAEIEGE+GDSHMGL ARM+SQAMRKL
RecAVch   119  DTGEQALEICDALARSGAVDVIVVDSVAALTPKAEIEGEMGDSHMGLQARMLSQAMRKLT  178

RecAEc    181  GNLKQSNTLLIFINQIRMKIGVMFGNPETTTGGNALKFYASVRLDIRRIGAVKEGENVVG  240
               GNLKQSN + IFINQIRMKIGVMFGNPETTTGGNALKFYASVRLDIRR GA+KEGE VVG
RecAVch   179  GNLKQSNCMCIFINQIRMKIGVMFGNPETTTGGNALKFYASVRLDIRRTGAIKEGEEVVG  238

RecAEc    241  SETRVKVVKNKIAAPFKQAEFQILYGEGINFYGELVDLGVKEKLIEKAGAWYSYKGEKIG  300
               +ETR+KVVKNKIAAPFK+A  QI+YG+G N  GEL+DLGVK K++EK+GAWYSY G+KIG
RecAVch   239  NETRIKVVKNKIAAPFKEANTQIMYGQGFNREGELIDLGVKHKMVEKSGAWYSYNGDKIG  298

RecAEc    301  QGKANATAWLKDNPETAKEIEKKVRELLLSNPNS---TPDFSVDDSEGVAETNEDF  353
               QGKANA  +LK+NPE AK ++KK+RE+LL+  N        s  D        E+F
RecAVch   299  QGKANACKYLKENPEIAKTLDKKLREMLLNPENMQLIAETSSAADDVEFGAVPEEF  354
```

Figure S2: **Alignment of the RecAEc and RecAVch protein sequences**
The alignment was made on Geneious. Identical amino acids are listed between the two sequences as well as functionally conservative substitution (shown by +).

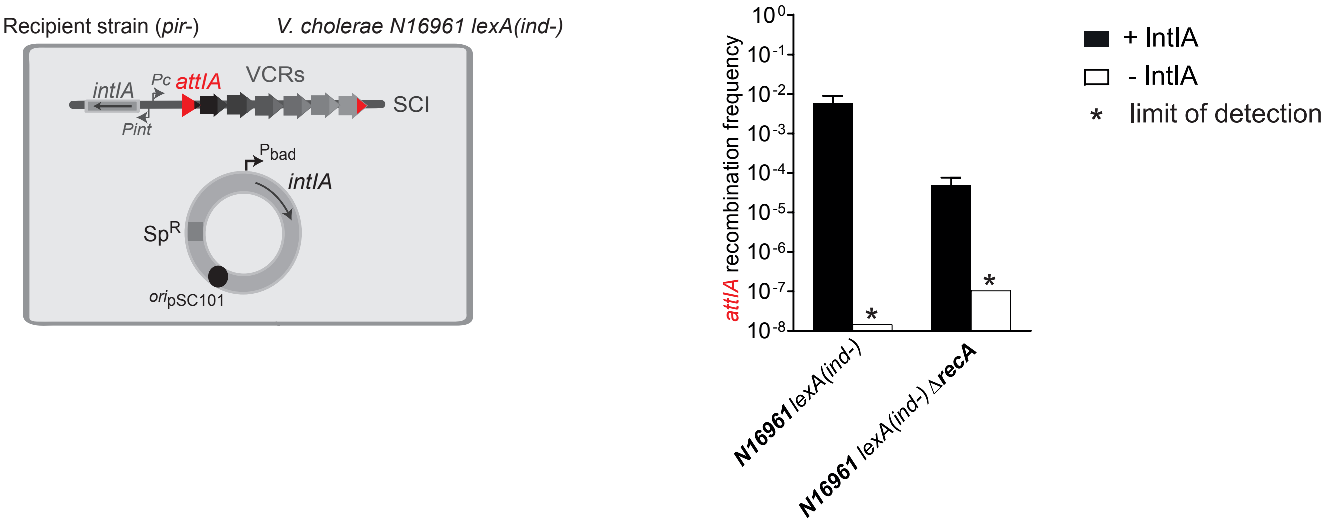

Figure S3: Effect of the RecA protein on *attIA* × *attC* recombination in the *V. cholerae lexA(ind-) strain*
Experimental setup and frequency of insertion of the pSW23T::$attC_{aadA7}$ suicide vector into the *attIA* site of the SCI. N16961 *lexA(ind-)* recipient strains transformed with the pBAD43 IntIA expressing vector were used (left panel). The recombination rates were calculated in N16961 *V. cholerae lexA(ind-)* and in the corresponding *recA* mutant strains (*lexA(ind-)ΔrecA*, right panel).

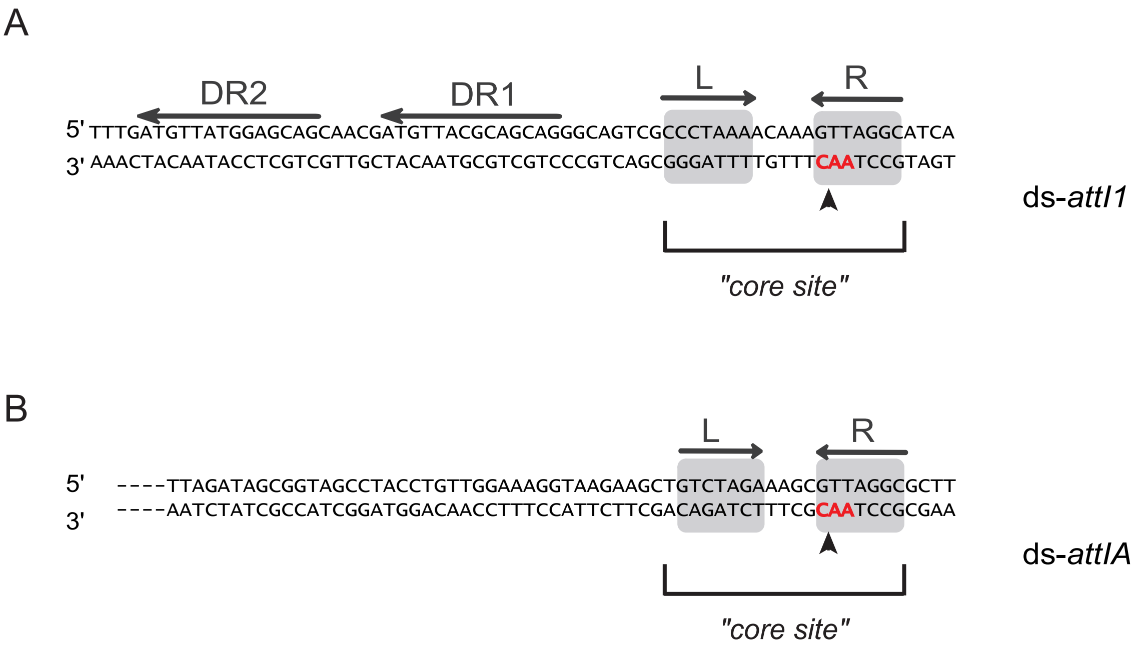

Figure S4: Schematic representation of the double-stranded *attI* recombination sites
The sequences of the double-stranded (ds) *attI* sites of the class 1 MI (A) and of the SCI of *Vibrio cholerae* platforms (B) are represented, respectively *attI1* and *attIA*. Grey boxes indicate the R (right) and L (left) integrase binding sites (core site). The 5'-AAC-3' triplet, where the cleavage takes place is highlighted in red and the precise cleavage point is indicated by a black arrowhead. The Direct Repeats (DR1 and DR2) of the *attI1* site are shown by black arrows.

Table S1: Bacterial strains used in this study

Strain number	Relevant genotypes or description	References
	**Basic *V. cholerae* strains**	
7805	N16961 biovar El Tor	Laboratory collection
8637	N16961 *hapR*+ [Str^R Gm^R]	Laboratory collection
9156	N16961 *hapR*+ *ΔintIA* [Str^R Gm^R]	(Leroux et al., 2007)
B306	7805 *ΔrecA*	This study, deletion of the *recA* gene (VC0543) was performed by allelic exchange by delivering the suicide conjugative plasmid pB203 in the 7805 strain.
H858	8637 *lexAind-* (A91D) [Str^R Gm^R]	This study, point mutation in *lexA* gene was introduced by allelic exchange by delivering the suicide conjugative plasmid p6780 (Guerin et al., 2009) in the 8637 strain.
P754	H858 *ΔrecA*	This study, deletion of the *recA* gene (VC0543) was performed by allelic exchange by delivering the suicide conjugative plasmid pB203 in the H858 strain.
L438	B306 *att*Tn7*::*P_{LAC}-$recA_{Ec}$	This study, P_{LAC}-recA$_{Ec}$ was inserted into the *att*Tn7 locus following the previously described protocol (de Lemos Martins et al., 2018) and by delivering by conjugation the pL310 shuttle vector into the B306 strain
L435-37	B306 *att*Tn7*::*P_{LAC}-$recA_{Vch}$	This study, P_{LAC}-recA$_{Vch}$ was inserted into the *att*Tn7 locus following the previously described protocol (de Lemos Martins et al., 2018) and by delivering by conjugation the pL312 shuttle vector into the B306 strain.

K779-K781	7805 *ΔattIA*	This study, *attIA* site was deleted by allelic exchange by delivering the suicide conjugative plasmid pK590 in the 7805 strain.
K782-K784	B306 *ΔattIA*	This study, *attIA* site was deleted by allelic exchange by delivering the suicide conjugative plasmid pK590 in the B306 strain that was previously transformed with the pAM::$recA_{Ec}$ vector.
K756-K757-K955	7805 *ΔattIA::attI1*	This study, *attIA* site was replaced by allelic exchange by delivering the suicide conjugative plasmid pK584 in the 7805 strain.
K956-K958	B306 *ΔattIA::attI1*	This study, *attIA* site was replaced by allelic exchange by delivering the suicide conjugative plasmid pK584 in the B306 strain that was previously transformed with the pAM::$recA_{Ec}$ vector.
	**Basic *E. coli* strains**	
8195	DH5α, (F-) *supE44 ΔlacU169* (φ80lacZΔM15) *ΔargF hsdR17 recA1 endA1 gyrA96 thi-1 relA1*	Laboratory Collection
4196	β2163, (F-) RP4-2-Tc::Mu *ΔdapA*::(*erm-pir*) [Km^R Em^R]	(Demarre et al., 2005)
8725	π3813, *lacIq thi-1 supE44 endA1 recA1 hsdR17 gyrA462 zei-298::Tn10 ΔthyA*::(*erm-pir116*) [Em^R]	(Le Roux et al., 2007)
8726	β3914, β2163 *gyrA462 zei-298::Tn10* [Km^R Em^R]	(Le Roux et al., 2007)
C349	MG1655 *E. coli* K12 wt	Laboratory collection
4826	MG1655 *recA269::Tn10* [Tc^R]	RG Lloyd

L805-L806	4826 *att*Tn7::P$_{LAC}$-*recA*$_{Vch}$ [Tc^R]	This study, P$_{LAC}$-recA$_{Vch}$ fragment was inserted into the *att*Tn7 locus following the previously described protocol (de Lemos Martins et al., 2018) by delivering the pL312 shuttle vector into the 4826 strain

	**Transformed strains of *V. cholerae* used in suicide conjugation assay**	
J554-J556	N16961 pJ502	This Study
J529-J531	N16961 pJ504	This Study
J566-J568	N16961 *hapR*+ pJ502	This Study
J541-J543	N16961 *hapR*+ pJ504	This Study
o150-o152	N16961 *hapR*+ *ΔintIA* pJ502	This Study
o153-o155	N16961 *hapR*+ *ΔintIA* pJ504	This Study
J557-J559	N16961*ΔrecA* pJ502	This Study
J532-J534	N16961*ΔrecA* pJ504	This Study
J569-J571	N16961 *hapR*+ *lexAind-* pJ502	This Study
J544-J546	N16961 *hapR*+ *lexAind-* pJ504	This Study
P771-P773	N16961 *hapR*+ *lexAind- ΔrecA* pJ502	This Study
P772-P774	N16961 *hapR*+ *lexAind- ΔrecA* pJ504	This Study
L453	N16961*ΔrecA att*Tn7::P$_{LAC}$-*recA*$_{Ec}$ pJ502	This Study
L447	N16961*ΔrecA att*Tn7::P$_{LAC}$-*recA*$_{Ec}$ pJ504	This Study
L449-L451	N16961*ΔrecA att*Tn7::P$_{LAC}$-*recA*$_{Vch}$ pJ502	This Study
L443-L445	N16961*ΔrecA att*Tn7::P$_{LAC}$-*recA*$_{Vch}$ pJ504	This Study
M933	N16961*ΔattIA* pJ502 pM923	This Study
M927	N16961*ΔattIA* pJ504 pM923	This Study
N457-N459	N16961*ΔrecA ΔattIA* pJ502 pM923	This Study
N454-N456	N16961*ΔrecA ΔattIA* pJ504 pM923	This Study
K870-K872	N16961*ΔattIA* pJ502	This Study
K864-K866	N16961*ΔattIA* pJ504	This Study
K873-K875	N16961*ΔrecA ΔattIA* pJ502	This Study

K867-K869	N16961*ΔrecA ΔattIA* pJ504	This Study
L326-L328	N16961*ΔattIA* p2597	This Study
L329-L331	N16961*ΔattIA* p8532	This Study
L338-L340	N16961*ΔrecA ΔattIA* p2597	This Study
L341-L343	N16961*ΔrecA ΔattIA* p8532	This Study
K976-K978	N16961*ΔattIA::attI1* pL290	This Study
K970-K972	N16961*ΔattIA::attI1* pL294	This Study
K979-K981	N16961*ΔrecA ΔattIA::attI1* pL290	This Study
K973-K975	N16961*ΔrecA ΔattIA::attI1* pL294	This Study
L581-L583	N16961*ΔattIA::attI1* p929 pL290	This Study
L584-L586	N16961*ΔattIA::attI1* p929 pL294	This Study
L713-L715	N16961*ΔrecA ΔattIA* p929 pL290	This Study
L716-L718	N16961*ΔrecA ΔattIA* p929 pL294	This Study
	**Transformed strains of *V. cholerae* used in suicide natural transformation assay**	
N389-N390	N16961 *hapR*+ pM889	This Study
N387-N388	N16961 *hapR*+ pN346	This Study
N535-N537	N16961 *hapR*+ *ΔintIA* pM889	This Study
N532-N534	N16961 *hapR*+ *ΔintIA* pN346	This Study

	**Transformed strains of *E. coli* in suicide conjugation assay**	
L120	β2163 pD060	This study
L385-L387	MG1655 p929 pL290	This study
J733-J735	MG1655 p929 pL294	This study
L388-L390	MG1655 p1105 pL290	This study
K689-K690	MG1655 p1105 pL294	This study
N369-N371	MG1655 pM923 pJ502	This study
N372-N374	MG1655 pM923 pJ504	This study
K686-K688	MG1655 p1105 pJ502	This study
K683-K685	MG1655 p1105 pJ504	This study

L391-L393	MG1655 *recA269::Tn10* p929 pL290	This study
J990-J991	MG1655 *recA269::Tn10* p929 pL294	This study
L394-L396	MG1655 *recA269::Tn10* p1105 pL290	This study
K698-K699-J992	MG1655 *recA269::Tn10* p1105 pL294	This study
N375-N377	MG1655 *recA269::Tn10* pM923 pJ502	This study
N378-N380	MG1655 *recA269::Tn10* pM923 pJ504	This study
K695-K697	MG1655 *recA269::Tn10* p1105 pJ502	This study
K692-K694	MG1655 *recA269::Tn10* p1105 pJ504	This study
N381-N383	MG1655 *recA269::Tn10*- *att*Tn7::P_{LAC}-*recA*$_{Vch}$ pM923 pJ502	This study
N384-N386	MG1655 *recA269::Tn10*- *att*Tn7::P_{LAC}-*recA*$_{Vch}$ pM923 pJ504	This study

	**Transformed strains of *V. cholerae* used in recombination assay with unidirectional-replicative substrate**	
K160-K162	N16961 p979 p7523	This study
K169-K171	N16961 p979 p7546	This study
K172-K174	N16961 p995 p7523	This study
K181-K183	N16961 p995 p7546	This study
K184-K186	N16961*ΔrecA* p979 p7523	This study
K193-K195	N16961*ΔrecA* p979 p7546	This study
K196-K198	N16961*ΔrecA* p995 p7523	This study
K205-K207	N16961*ΔrecA* p995 p7546	This study

Table S2: Plasmids used in this study

Plasmid number	Plasmid description	Relevant properties and construction

p452	pSU18Δ::*VCR*$_{128}$	*ori*p15A; [Cm^R] (Biskri et al., 2005)
p741	pSU38Δ	*ori*p15A; [Km^R] (Biskri et al., 2005)
p755	pSU38Δ::*attIA*	*ori*p15A; [Km^R] (Biskri et al., 2005)
p929	pSU38Δ::*attI1*	*ori*p15A; [Km^R] (Biskri et al., 2005)
p1105	pSU38Δ::VCR$_{VCA0441}$	*ori*p15A; [Km^R] EcoRI/BamHI fragment (VCR) from p452 cloned in EcoRI/BamHI digested p741 (Biskri, unpublished results)
p7523	pTSC29::*attC*$_{aadA7}$ (ori-) ("lead")	*oripSC101*ts; [Cm^R] (Loot et al., 2010)
p7546	pTSC29::*attC*$_{aadA7}$ (ori+) ("lag")	*oripSC101*ts; [Cm^R] (Loot et al., 2010)
p979	pBAD18	*ori*ColE1; [Carb^R] (Guzman et al., 1995)
p995	pBAD18::*intIA*$_{Vch}$	*ori*ColE1; [Carb^R] (Biskri et al., 2005)
p3938	pBAD18::*intI1*	*ori*ColE1; [Carb^R] (Demarre et al., 2007)
p2597	pBAD43	*oripSC101*; [Sp^R] (Guzman et al., 1995)
p8532	pBAD43::*intIA*$_{Vch}$	*oripSC101*; [Sp^R] (Biskri, unpublished)
pG906	pBAD43::*intI1*	*oripSC101*; [Sp^R] EcoRI/HindIII digested fragment (*intI1*) from p3938 cloned in EcoRI/HindIII digested p2597
pJ502	pBAD43*ΔattC*$_{aadA1}$	*oripSC101*; [Sp^R] amplification by inverse PCR of p2597 with primers 5640 and 5641
pJ504	pBAD43 *ΔattC*$_{aadA1}$::*intIA*$_{Vch}$	*oripSC101*; [Sp^R] amplification by inverse PCR of p8532 with primers 5640 and 5641
pL290	pBAD43::*aadA7*	*oripSC101*; [Sp^R] The fragment P$_{cat}$-*aadA7* was amplified from pSW25T vector (Demarre et al., 2005) with primers 5234 and 5235. pJ502 vector was amplified by inverse PCR with 5914 and 5915 primers. Assembly of these two fragments was achieved by performing Gibson Assembly.
pL294	pBAD43::*aadA7-intI1*	*oripSC101*; [Sp^R] The same strategy was used as for the construction of pL290 but using pG906 as template for vector amplification with 5914 and 5915 primers.

pM783	pSU38Δ*::intIA-attIA*	*ori*p15A; [Km^R] The *intIA-attIA* fragment was amplified from the 7805 strain using o6176 and o6177 primers. This fragment was digested with EcoRI and BamHI restriction enzymes and ligated into the EcoRI and BamHI digested p755 vector.
pM923	pSU38Δ*::intIAY302F-attIA*	*ori*p15A; [Km^R] Inverse PCR was performed using the pM783 plasmid and, o6184 and o6185 primers.
pE639	pLC10	*oripSC101ts*; *oripSC101*; [Sp^R] Laboratory of David Bikard, Institut Pasteur
p7301	pCY579 (pAM*::*$recA_{Ec}$)	*oripSC101*; [$Carb^R$] (Cronan, 2003)
pM889	pBAD43$\Delta attC_{aadA1}$ -P_{tet}	*oripSC101*; [Sp^R] The P_{tet} promoter fragment was amplified from pE639 vector using 6178 and 6182 primers. pJ502 vector was amplified by inverse PCR with 6181 and 6183 primers. Assembly of these two fragments was achieved by performing Gibson Assembly.
pN346	pBAD43$\Delta attC_{aadA1}$-P_{tet}-*intIA*	*oripSC101*; [Sp^R] The P_{tet} promoter fragment was amplified from pE639 vector using 6178 and 6230 primers. pJ504 vector was amplified by inverse PCR with 6181 and 6229 primers. Assembly of these two fragments was achieved by performing Gibson Assembly.
pD060	pSW23T*::*$attC_{aadA7}$ (bs)	$oriV_{R6K\gamma}$, $oriT_{RP4}$; [Cm^R] (Nivina et al., 2016)
p7848 (pMP7)	Suicide conjugative plasmid used for allelic exchange pSW23T-*araC* P_{BAD}-*ccdB*	$oriV_{R6K\gamma}$, $oriT_{RP4}$; [Cm^R] (Val et al., 2012)
p6780	Suicide conjugative plasmid used for replacement of *lexAind-* allele	$oriV_{R6K\gamma}$, $oriT_{RP4}$; [Cm^R] (Guerin et al., 2009)

pB203	Suicide conjugative plasmid used for deletion of *recA*$_{Vch}$ gene (VC0543)	*oriV*$_{R6K\gamma}$, *oriT*$_{RP4}$; [Cm^R] homology regions upstream and downstream of the VC0543 gene were amplified with VC0542A and RECAB and with RECAC and RECAD primers respectively. For both PCR the N16961 genomic DNA was used as a template. These two fragments were assembled by PCR using VC0542A and RECAD primers. This combined fragment was digested by EcoRI and cloned into EcoRI digested p7848 vector. (Krin, unpublished)
pK584	Suicide conjugative plasmid used for deletion of *attIA* and replacement by *attI1*	*oriV*$_{R6K\gamma}$, *oriT*$_{RP4}$; [Cm^R] amplification of fragments upstream and downstream of *attIA* were performed using 5738 and 5988 and, 5744 and 5739 respectively. Both PCR were performed using N16961 genomic DNA as template. These two fragments were assembled by PCR using 5738 and 5739 primers. The obtained fragment was then digested by EcoRI/PstI and cloned into EcoRI/PstI digested p7848 vector.
pK590	Suicide conjugative plasmid used for deletion of *attIA*	*oriV*$_{R6K\gamma}$, *oriT*$_{RP4}$; [Cm^R] amplification by inverse PCR of pK584 plasmid with primers 5761 and 5762.
pH996 (pMP234)	Tn7 shuttle vector: pSW23T*::* [Tn7R-MCS-Tn7L]	*oriV*$_{R6K\gamma}$, *oriT*$_{RP4}$; [Cm^R] (Val, unpublished)
pA684	pUC18*::recA*$_{Vch}$	*ori*ColE1; [Carb^R] amplification of *recA*$_{Vch}$ fragment by PCR using recAeco5 and recApst3 primers and N16961 genomic DNA as template. This fragment was then digested with EcoRI/PstI and cloned into EcoRI/PstI digested pUC18 (Pharmacia) vector (Krin, unpublished)
pK621	pUC18*::recA*$_{Ec}$	*ori*ColE1; [Carb^R] amplification of *recA*$_{Ec}$ fragment by PCR using 5779 and 5780 primers and MG1655 genomic DNA as template. This fragment was

		cloned by Gibson Assembly into EcoRI/PstI digested pUC18.
pL122	pUC18*ΔlacZα::recA*$_{Vch}$	*ori*ColE1; [Carb^R] amplification by inverse PCR of the pA684 plasmid with 5799 and 5800 primers.
pL123	pUC18 *ΔlacZα::recA*$_{Ec}$	*ori*ColE1; [Carb^R] amplification by reverse PCR of pA684 plasmid with VN9 and 5800 primers.
p7741	pKD4	*oriV*$_{R6K\gamma}$; [Carb^R, Km^R] (Datsenko and Wanner, 2000).
pL310	pMP234::P$_{LAC}$-*recA*$_{EC}$-FRT-*aph*-FRT	*oriV*$_{R6K\gamma}$, *oriT*$_{RP4}$; [Cm^R] the P$_{LAC}$-*recA*$_{Ec}$ fragment was amplified with 5918-5916 primers from pL123 vector and the FRT-*aph*-FRT fragment was amplified with 5917-5919 primers from p7741 vector. These fragments were assembled by PCR using 5918 and 5919 primer. The obtained fragment was cloned by Gibson Assembly into pMP234 vector backbone that was amplified by inverse PCR with primers 5480 and 5481.
pL312	pMP234::P$_{LAC}$-*recA*$_{Vch}$-FRT-*aph*-FRT	*oriV*$_{R6K\gamma}$, *oriT*$_{RP4}$; [Cm^R] The same cloning procedure was used as for pL310 construction. However, in this case, the P$_{LAC}$-*recA*$_{Vch}$ fragment was amplified from the pL122 vector.
pF324 (pMVM1)	Tn7 helper: *araC* P$_{BAD}$-*tnsABCD*	*oripSC101*ts, *oriT*$_{RP4}$; [Carb^R] (de Lemos Martins et al., 2018)

Table S3: Primers used in this study

Primers	Sequences
	**Primers used for Plasmid construction**
recAeco5	GGAATTCGATGGACGAGAATAAACAGAA
recApst3	AACTGCAGTTAAAACTCTTCTGGCACCG
VN9	ATGGACGAGAATAAACAGAAGG

VC0542A	GGAATTCGCTTTGTGTTTGATTTCTTT
RECAB	CTTTGCATTCAGCCTGCCGATACTCTCTCCGGATAGTCAC
RECAC	GTGACTATCCGGAGAGAGTATCGGCAGGCTGAATGCAAAG
RECAD	GGAATTCGTGGCTGATGCCGCTTTTGA
5234	TTTCTAGGCACCAATAACTGCCTTA
5235	GTTGATACCGGGAAGCCCTGGGCCA
5480	GCACCTAGGAGGCGCGCCAC
5481	CGCTATTGACCCGGGATCTG
5640	GACATTATTTGCCGACTACCTTGGTGATCTCGC
5641	GATGCACTAAGCACATAATTGCTCACAGCC
5738	CGGGAATTCCCGTAGAGTAACTTGATGGGAAGTT
5739	CGGCTGCAGGAGGTCAAACTATAAAACACCCAAGC
5744	CCCTAAAACAAAGTTAGGCATCAGAGCTTTCTTGCTAATGTTAGATCAAT
5761	CTAATTAATAGACCACTGGGTGC
5762	GCTTTCTTGCTAATGTTAGATCA
5779	AGTGCCAAGCTTGCATGCCTGCAGTTAAAAATCTTCGTTAGTTTCTGC
5780	CAGCTATGACCATGATTACGAATTCGATGGCTATCGACGAAAACAAACAG
5799	ATGGCTATCGACGAAAACAAACAGAAAGC
5800	AGCTGTTTCCTGTGTGAAATTGTTATCC
5914	TAAGGCAGTTATTGGTGCCTAGAAAGTCGATGCACTAAGCACATAATTGC TCAC
5915	TGGCCCAGGGCTTCCCGGTATCAACAATTCCCACGGGTTTTGCTGCCCGC
5916	GCTCCAGCCTACACAATCGCTCAAAAACGACGGCCAGTGCCAAGCTTGC
5917	GCAAGCTTGGCACTGGCCGTCGTTTTTGAGCGATTGTGTAGGCTGGAGC
5918	CAGATCCCGGGTCAATAGCGACTGGAAAGCGGGCAGTGAGCGC
5919	GTGGCGCGCCTCCTAGGTGCATGGTCCATATGAATATCCTCC
5988	TGATGCCTAACTTTGTTTTAGGGCGACTGCCCTGCTGCGTAACATCGTTGC TGCTCCATAACATCAAACTAATTAATAGACCACTGGGTGC
6176	GGCCGAATTCCTGTGAAATCTCATGATTTCGC
6177	GGCCGGATCCGGATAGATGCATATAATTCGC
6184	TCTGTGTCGTTTTTACATCGGTATGTCC
6185	TTTTCACTCATGTTCTTGATAGAGGTGCAAGCG

6178	CCCTATGCTACTCCGTCAAGCCGTCAATTGTCTGATTCGTTACCAACACGC CTGCCAGGAATTGGGGATCGGTTAAGACCC
6181	GGGTCTTAACCGATCCCCAATTCCTGGCAGGCGTGTTGGTAACGAATCAG ACAATTGACGGCTTGACGGAGTAGCATAGGG
6182	CGCAGGGGTAGTGAATCCGCCAGGATTGACTTGCGCTGCCTTTTGCCTCCT AACTAGGTCATTTGATATGCCTCCGG
6183	CCGGAGGCATATCAAATGACCTAGTTAGGAGGCAAAAGGCAGCGCAAGT CAATCCTGGCGGATTCACTACCCCTGCG
6229	CCGGAGGCATATCAAATGACCTAGTTAGGAGGCAAAAATGAAATCCCAG TTTTTGTTAAGTGTTCGCGAATTTATGCAAACTCG
6230	CGAGTTTGCATAAATTCGCGAACACTTAACAAAAACTGGGATTTCATTTTT GCCTCCTAACTAGGTCATTTGATATGCCTCCGG
	**Primers used to identify the location of cassette insertion**
SWbeg	CCGTCACAGGTATTTATTCGGCG
SWend	CCTCACTAAAGGGAACAAAAGCTG
MFD	CGCCAGGGTTTTCCCAGTCAC
573	GCTGCCCGGATTACACC
1366	AGCGGGTGTTCCTTCTTCACTG
1388	CCGGGCAGGATAGGTGAAGTAG
1704	AGAGAACATAGCGTTGCCTTGG
1863	GGCCACGCGTCGACTAGTACNNNNNNNNNNACGCC
1865	GGCCACGCGTCGACTAGTAC
2405	ATACGACTCACTATAGGGCG
5778	GTCAAGAGGCTATACAGACATCAGC

References

Biskri, L., Bouvier, M., Guerout, A.M., Boisnard, S., and Mazel, D. (2005). Comparative Study of Class 1 Integron and *Vibrio cholerae* Superintegron Integrase Activities. J Bacteriol *187*, 1740-1750.

Cronan, J.E. (2003). Cosmid-based system for transient expression and absolute off-to-on transcriptional control of Escherichia coli genes. J Bacteriol *185*, 6522-6529.

Datsenko, K.A., and Wanner, B.L. (2000). One-step inactivation of chromosomal genes in Escherichia coli K-12 using PCR products. Proc Natl Acad Sci U S A *97*, 6640-6645.

de Lemos Martins, F., Fournes, F., Mazzuoli, M.V., Mazel, D., and Val, M.E. (2018). Vibrio cholerae chromosome 2 copy number is controlled by the methylation-independent binding of its monomeric initiator to the chromosome 1 crtS site. Nucleic Acids Res *46*, 10145-10156.

Demarre, G., Frumerie, C., Gopaul, D.N., and Mazel, D. (2007). Identification of key structural determinants of the IntI1 integron integrase that influence attC x attI1 recombination efficiency. Nucleic Acids Res *35*, 6475-6489.
Demarre, G., Guerout, A.M., Matsumoto-Mashimo, C., Rowe-Magnus, D.A., Marlière, P., and Mazel, D. (2005). A new family of mobilizable suicide plasmids based on the broad host range R388 plasmid (IncW) or RP4 plasmid (IncPα) conjugative machineries and their cognate *E.coli* host strains. Research in Microbiology *156*, 245-255.
Guerin, E., Cambray, G., Sanchez-Alberola, N., Campoy, S., Erill, I., Da Re, S., Gonzalez-Zorn, B., Barbe, J., Ploy, M.C., and Mazel, D. (2009). The SOS response controls integron recombination. Science *324*, 1034.
Guzman, L.M., Belin, D., Carson, M.J., and Beckwith, J. (1995). Tight regulation, modulation, and high-level expression by vectors containing the arabinose PBAD promoter. J Bacteriol *177*, 4121-4130.
Loot, C., Bikard, D., Rachlin, A., and Mazel, D. (2010). Cellular pathways controlling integron cassette site folding. EMBO J *29*, 2623-2634.
Nivina, A., Escudero, J.A., Vit, C., Mazel, D., and Loot, C. (2016). Efficiency of integron cassette insertion in correct orientation is ensured by the interplay of the three unpaired features of attC recombination sites. Nucleic Acids Res *44*, 7792-7803.
Val, M.E., Skovgaard, O., Ducos-Galand, M., Bland, M.J., and Mazel, D. (2012). Genome engineering in Vibrio cholerae: a feasible approach to address biological issues. PLoS Genet *8*, e1002472.
